# Non-canonical telomere protection role of FOXO3a of human skeletal muscle cells regulated by the TRF2-redox axis

**DOI:** 10.1038/s42003-023-04903-1

**Published:** 2023-05-25

**Authors:** Maria Sol Jacome Burbano, Jérôme D. Robin, Serge Bauwens, Marjorie Martin, Emma Donati, Lucia Martínez, Peipei Lin, Sabrina Sacconi, Frédérique Magdinier, Eric Gilson

**Affiliations:** 1 Université Côte d’Azur, CNRS, Inserm, IRCAN, Faculté de médecine Nice, Nice, France; 2 Department of Geriatrics, Medical center on Aging of Shanghai Ruijin Hospital, Shanghai Jiaotong University School of Medicine, Shanghai, China; 3 Pôle Sino-Français de Recherches en Sciences du Vivant et Génomique, International Research Project in Hematology, Cancer and Aging, RuiJin Hospital, Shanghai Jiao Tong University School, Shanghai, China; 4 Peripheral Nervous System, Muscle and ALS, Neuromuscular & ALS Center of Reference, FHU Oncoage, Nice University Hospital, Pasteur 2, Nice, France; 5 Aix Marseille Univ, INSERM, MMG, 13005 Marseille, France; 6 Department of Genetics, CHU; FHU OncoAge, Nice, France

**Keywords:** Cell biology, Molecular biology

## Abstract

Telomeric repeat binding factor 2 (TRF2) binds to telomeres and protects chromosome ends against the DNA damage response and senescence. Although the expression of TRF2 is downregulated upon cellular senescence and in various aging tissues, including skeletal muscle tissues, very little is known about the contribution of this decline to aging. We previously showed that TRF2 loss in myofibers does not trigger telomere deprotection but mitochondrial dysfunction leading to an increased level of reactive oxygen species. We show here that this oxidative stress triggers the binding of FOXO3a to telomeres where it protects against ATM activation, revealing a previously unrecognized telomere protective function of FOXO3a, to the best of our knowledge. We further showed in transformed fibroblasts and myotubes that the telomere properties of FOXO3a are dependent on the C-terminal segment of its CR2 domain (CR2C) but independent of its Forkhead DNA binding domain and of its CR3 transactivation domain. We propose that these non-canonical properties of FOXO3a at telomeres play a role downstream of the mitochondrial signaling induced by TRF2 downregulation to regulate skeletal muscle homeostasis and aging.

## Abstract (web summary)

Loss of the telomeric repeat binding factor 2 (TRF2) and its resulting oxidative stress leads to FOXO3a binding to telomeres, which protects against activation of ATM to maintain skeletal muscle homeostasis.

## Introduction

Aging can be defined as a progressive failure of homeostasis and organ function that increases susceptibility to several diseases and limits longevity^1^. An extraordinary diversity of aging trajectories exists in nature^2^, which can be explained by the effects of natural selection aimed at maximizing fitness in a given environmental context^3^. Mechanistically, aging is caused by limitation of somatic maintenance and stress response mechanisms, leading to a gradual increase in molecular and cellular damage along with senescent cell accumulation^4,5^. The rate of deterioration is determined by interconnected biological processes, known as aging hallmarks, which are regulated by environmental and genetic factors^6^. Pro-longevity genes, protecting against aging hallmarks, have been identified in genome-wide association studies. For example, several studies have consistently identified FOXO3a as a pro-longevity master transcription factor that serves as a stress sensor and homeostasis regulator^7,8^.

The existence of one or several developmentally regulated clocks that orchestrate the aging hallmarks remains unclear. In human and other vertebrates, telomeres may represent such an aging clock, because their DNA length is programmed to shorten in somatic cells during embryonic development^9,10^ and upon stress exposure, accounting in part for the disparate trajectories of aging^11,12^. Moreover, dysregulation of telomeres is associated with rare progeroid syndromes^13^ and a broad spectrum of age-related pathologies in the general population^14^, whereas their reinforcement extends the lifespan in mouse models^15,16^. This central position of telomeres as an aging regulator might stem from their interconnection to several aging hallmarks such as genome stability^17^, senescence^18^, oxidative stress^19^, and mitochondrial integrity^20,21^.

Many telomere functions are performed by a capping protein complex called shelterin, which consists of proteins directly bound to telomeric DNA (in Human, TRF1, TRF2, and POT1) and other proteins that create a bridge between the duplex DNA and the 3′ overhang (in Human, TPP1/ACD and TIN2) or are simply bound to other subunits (in humans, RAP1 binds to TRF2). Among shelterin subunits, TRF2 emerges as an important regulatory functions during aging, as it interconnects various aging hallmarks (genome stability, senescence, mitochondria, heterochromatin, and immunity), is downregulated during aging in multiple tissues^21,22^, and plays pivotal roles in aging in model organisms^23–26^. The multiple roles of TRF2 rely on its ability to blunt the DNA damage response (DDR) ataxia–telangiectasia mutated (ATM) checkpoint and non-homologous DNA repair at chromosomal termini, in addition to genome-wide transcriptional functions^27^ and heterochromatin replication^28^. For instance, TRF2 regulates the expression of glycocalyx genes involved in immunosurveillance^29^, the *SIRT3* sirtuin gene required for mitochondrial integrity^21^ and the *hTERT* telomerase gene^30,31^.

Strikingly, in skeletal muscle fibers, the absence of TRF2 does not lead to detectable telomere damage, suggesting that an alternative telomere protective mechanism exists in these long-lived post-mitotic cells^21^. Here, we demonstrate that FOXO3a binds to and protects the telomeres of skeletal muscle cells upon *TERF2* downregulation. This role of FOXO3a in telomere protection is also at play in dividing cells upon genotoxic stress. These results reveal a direct connection between telomere protection and Foxo3a activity, two key longevity pathways.

## Results

### Antioxidant treatment induces telomeric damage in *TERF2*-compromised myotubes

**Fig. 1:**
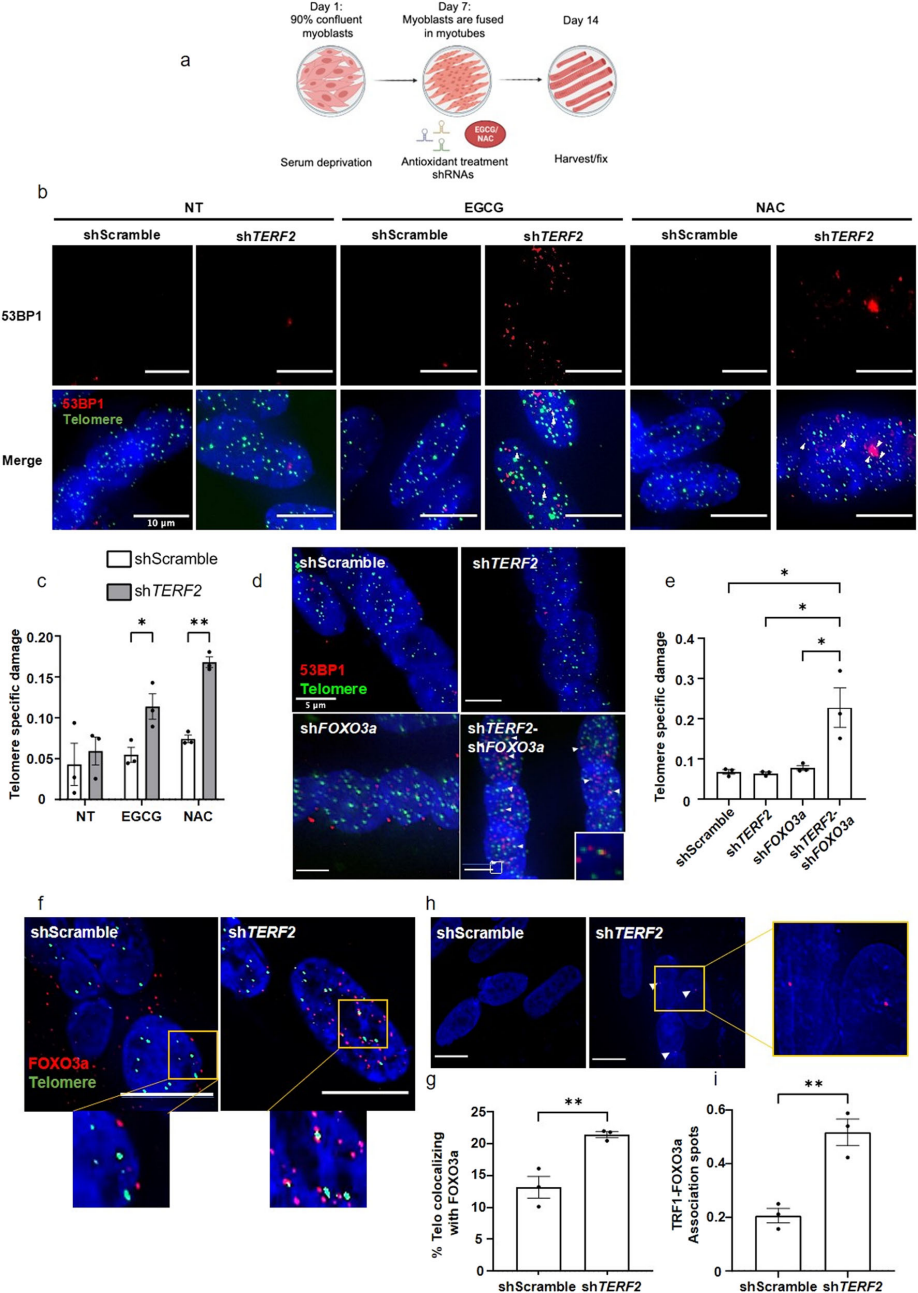
FOXO3a is activated by increased ROS and directly protects telomeres in myotubes depleted for *TERF2*. **a** Schematic representation of the strategy used, created using BioRender.com. Human myoblasts started differentiation into myotubes by serum deprivation on day 1. On day 7, most myoblasts are fused into multinuclear tubules. At this point, cells are transduced with shRNAs (shScramble and sh*TERF2*) and treated with antioxidants (ECGC or NAC). Myotubes are either fixed or harvested on day 14. **b** Telomere Induced damage Foci (TIF) (PNA-FISH) assay in shScramble or sh*TERF2* human myotubes (10 days), treated with antioxidants (EGCG and NAC) for 10 days. Telomeric PNA probe (green) and 53BP1 (red) staining. Colocalizations are pointed by a white arrow. Quantification in (**c**) Telomeric specific damage: ratio between the number of TIFs and the number of 53BP1 foci. Conditions were compared by two-way ANOVA, followed by Tukey’s post hoc test, (*p < 0.05, **p < 0,001). **d** TIF assay in transduced myotubes (10 days), using a telomeric PNA probe (green) and 53BP1 (red) staining. Colocalizations pointed by an arrow, indicate DDR activation at telomeres. 30–40 nuclei were analyzed per replicate and per condition. **e** Specific telomere damage (sTIF) of images in (**d**). Statistical analyses were calculated using one-way ANOVA, followed by Tukey’s post hoc test (*p < 0.05), 30–40 nuclei were analyzed per replicate and per condition, *n* = 3. **f** Immunofluorescence detection of FOXO3a (red), combined with telomeric FISH probe (green) in myotubes transduced with sh*TERF2*. Scale bar 8 μm. Quantification of the percentage of telomeres colocalizing with FOXO3 (**g**). Statistical analyses were performed using an unpaired *t*-test, *n* = 3, *p-value:* 0,0099. **h** Proximity Ligation Assay (PLA) between TRF1 and FOXO3a (red spots) in myotubes downregulated for *TERF2*. White arrows point association spots. Scale bar 10 μm. **i** Quantification of three biological replicates. Results were compared by an unpaired t-test, *p-value* = 0,0051. Mean ± SEM (error bars).

Previously, we showed that *TERF2* knockdown (KD) in human myotubes did not trigger DDR at telomeres but led to mitochondrial dysfunction and a high level of reactive oxygen species (ROS)^21^. To determine whether this oxidative stress impacted telomere functions, we treated myotubes with two antioxidants that we previously shown capable to blunt the ROS augmentation induced by TRF2 KD in the same myotube setting^21^: N-acetylcysteine (NAC) and epigallocatechin gallate (EGCG) (Fig. 1a). To distinguish between a global DNA damage response and specific damage to telomeres, we expressed telomere damage as the ratio of 53BP1 foci associated with telomeres to the total number of 53BP1 foci, a value designated as telomere specific damage (sTIF) hereafter. Astonishingly, upon *TERF2* downregulation, these antioxidants increased the rate of sTIF (Fig. 1b, c; Supplementary Fig. 1a, b). We conclude that a mechanism activated by oxidative stress triggers specific telomere protection in sh*TERF2*-transduced myotubes.

### FOXO3a is required for telomere protection upon *TERF2* downregulation in myotubes

*TERF2* downregulation in myotubes activates the longevity factor FOXO3a, as assayed by its nuclear translocation, likely as a result of mitochondrial dysfunction and elevated ROS levels^21^. Therefore, we investigated whether FOXO3a is involved in the specific telomere-protective effect of oxidative stress in *TERF2*-compromised myotubes. The downregulation of *FOXO3a* expression did not alter telomere protection in myotubes but synergized with *TERF2* KD to increase the rate of sTIFs (Fig. 1d, e; Supplementary Fig. 1c–e). Further, treatment with chemical inhibitors of the DDR kinases ATM (KU-55933) and ATR (ATR; VE-821) revealed that the telomere-specific damages triggered by double downregulation of *TERF2* and *FOXO3a* in myotubes were ATM dependent (Supplementary Fig. 1f).

Next, we asked whether the telomere-protective function of FOXO3a in *TERF2* KD myotubes can be explained by the antioxidant activities of FOXO3a. However, downregulation of *FOXO3a* expression did not further increase the elevated ROS level induced by *TERF2* downregulation (Supplementary Fig. 2a, b). Moreover, we studied the expression of a well-known panel of oxidative stress-related genes (e.g., *NRF2*, *GSS*, *BNIP3*, *PRDX1*, *SOD2*, and *CAT*). Among them, *CAT* and *SOD2* were downregulated upon *FOXO3A* KD but not further repressed in myotubes doubly downregulated for *TERF2* and *FOXO3a* (Supplementary Fig. 2c–h), which does not make it possible to explain the effect of the deprotection effect of the telomeres of the double KD. Overall, it appeared unlikely that the telomere deprotection triggered by the double downregulation of *FOXO3a* and *TERF2* can be caused by a global increase in ROS or a decrease in oxidative defense.

We conclude that FOXO3a impairs ATM-dependent DDR activation at the telomeres of myotubes with downregulated *TERF2* expression independently of its canonical function as an antioxidant regulator.

### FOXO3a localizes to telomeres of skeletal muscle cells

Next, we investigated whether FOXO3a can associate with telomeres. Roughly 12% of telomeres colocalized with FOXO3a foci in myotube nuclei, as detected by confocal microscopy using a telomeric PNA probe (Fig. 1f, g). Upon *TERF2* downregulation, in parallel with nuclear translocation of activated FOXO3a (Supplementary Fig. 3a), we measured an increase to 21% in the percentage of telomeres colocalizing with FOXO3a (Fig. 1f, g). These colocalizations correspond to a close association between telomere and FOXO3a, as further confirmed by proximity ligation assay (PLA) using TRF1 and FOXO3a as potential partners (Fig. 1h, i; Supplementary Fig. 1g, h) and chromatin immunoprecipitation (ChIP) with slot-blotting quantification using a radiolabeled telomeric DNA probe (Supplementary Fig. 3b, c). These results indicate that FOXO3a plays a direct role in telomere protection in myotubes upon *TERF2* downregulation.

Then, we investigated the interaction between telomere and FOXO3a during muscle aging using a set of human muscle biopsies^21^. In these biopsies, the presence of FOXO3a at telomeres was assayed by co-immunoprecipitation (co-IP) between TRF2 and FOXO3a (Supplementary Fig. 3d–f). Notably, the level of co-IP increased with age (Supplementary Fig. 3f) while the level of TRF2 decreased^21^. We interpret these seemingly contradictory results as an important increase in telomere–FOXO3a co-IP with age that can still be detected by co-IP with the residual amount of TRF2. Overall, these results indicate that FOXO3a associates with telomeres in human skeletal muscle, which increases with age.

### FOXO3a acts synergistically with TRF2 to protect telomeres in transformed fibroblasts

**Fig. 2:**
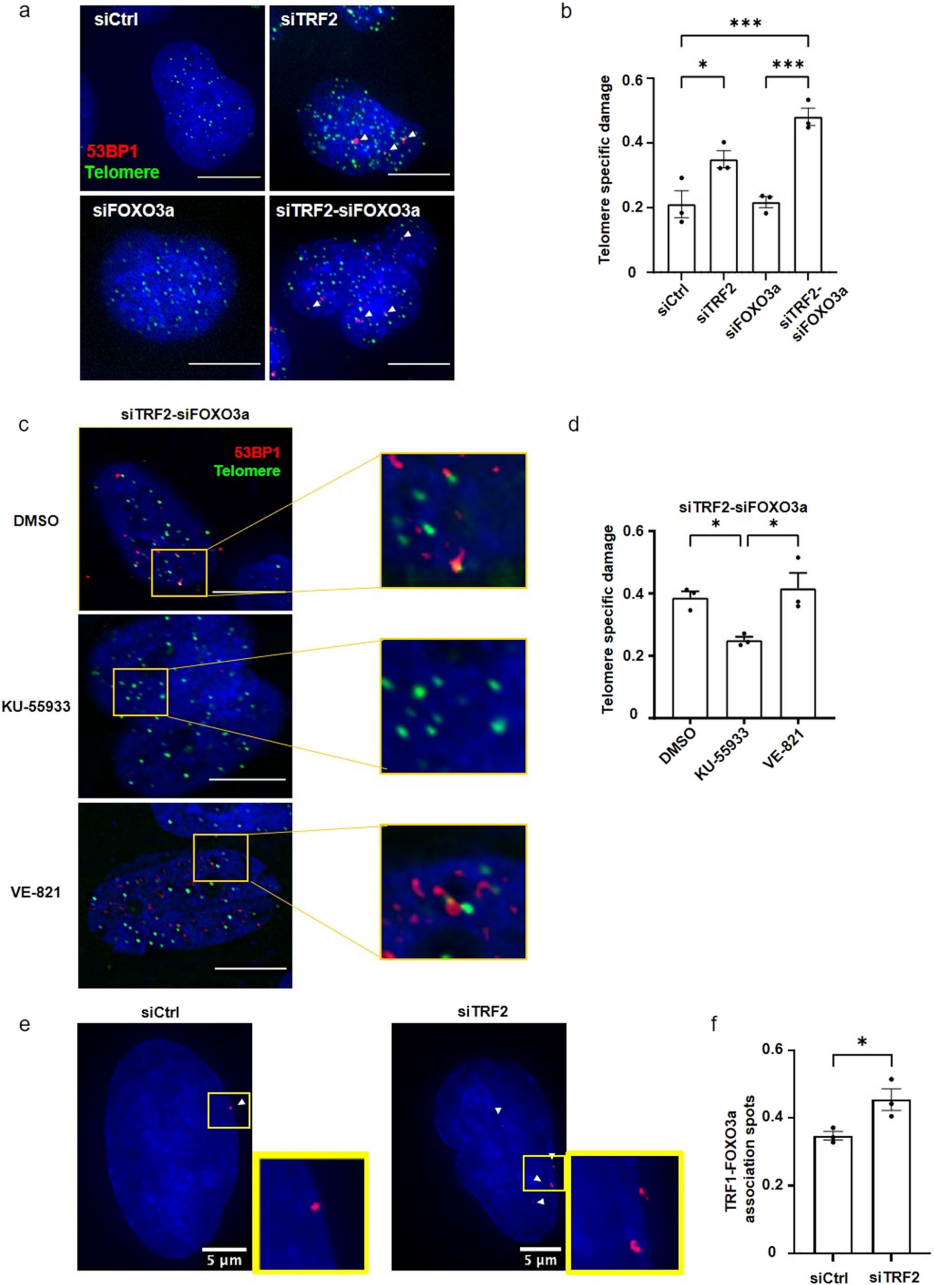
FOXO3a protects telomeres in BJ-HELT fibroblasts downregulated for TRF2. **a** Telomere Induced damage Foci assay in transfected BJ-HELT (72 h siRNA), using a telomeric PNA probe (green) and 53BP1 (red) staining. 53BP1-Telomeres colocalizations are pointed by a white arrow. Scale bar 10 μm. **b** Quantification of Telomere specific damage (sTIF) observed in (**a**). 30–40 nuclei were analyzed in three biological replicates. Statistical significance was calculated with a one-way ANOVA (*p < 0.05, ***p < 0.001). **c** TIF assay (telomeric PNA probe in green and 53BP1 in red) in BJ-HELT cells downregulated for TRF2 and FOXO3a and inhibited for ATM or ATR with KU-55933 and VE-821, respectively. Scale bar 10 μm. Quantified in (**d**), *n* = 3, *p < 0.05 using a one-way ANOVA test with Tukey’s post hoc test. **e** PLA TRF1-FOXO3a in BJ-HELT cells transfected with siCtrl or siTRF2. FOXO3a-TRF1 association spots are stained in red and are shown with a white arrow. A zoom of a representative area is on the right. **f** PLA TRF1-FOXO3a quantification. A two-tailed t-test showed a slight but significant increase in the siTRF2, *p-value* = 0,0319. Error bars indicate SEM.

Next, we investigated whether the telomeric-protection function of FOXO3a was replicated in dividing cells. To this end, we used BJ-HELT transformed fibroblasts derived from human foreskin cells expressing *hTERT* and *SV40 early region* (large-T and small-t antigens) genes. In these cells, downregulation of *FOXO3a* did not lead to specific telomere damage, whereas *TERF2* downregulation did, as previously reported^32–40^ (Fig. 2a, b; Supp Fig. 4a, b). Notably, concomitant downregulation of *FOXO3a* and *TERF2* showed synergistic effects, exacerbating the rate of sTIFs (Fig. 2a, b) in an ATM-dependent manner (Fig. 2c, d). Moreover, *TERF2* downregulation slightly increased TRF1-FOXO3a association, as determined by PLA (Fig. 2e, f and Supplementary Fig. 4c). This situation is reminiscent of myotubes because the telomere-protective function of FOXO3a against ATM is induced by *TERF2* downregulation. However, in contrast to myotubes, we failed to detect a difference in FOXO3a expression or nuclear translocation upon *TERF2* downregulation in BJ-HELT cells (Supplementary Fig. 4d, e). By undefined reasons, FOXO3a seems to be importantly activated in these cells, even in basal or unchallenged conditions. Next, we asked whether the telomere protection conferred by FOXO3a is also induced by downregulation of another shelterin subunit. Thus, we downregulated the six shelterin subunits with and without *FOXO3a* downregulation (Supplementary Fig. 4f, g). Only the downregulation o*f FOXO3a* in *TERF2-*compromised cells increased the level of telomere damage (Supplementary Fig. 4h). Since TRF2 is the shelterin subunit specifically involved in blunting ATM activation^41^, this result is in accordance with the ATM-dependent damage protected by *FOXO3a* in *TERF2-*downregulated cells (Supplementary Fig. 1f in myotubes and Supplementary Fig. 3c, d in BJ-HELT cells).

### FOXO3a protects telomeres from bleomycin induced damage

**Fig. 3:**
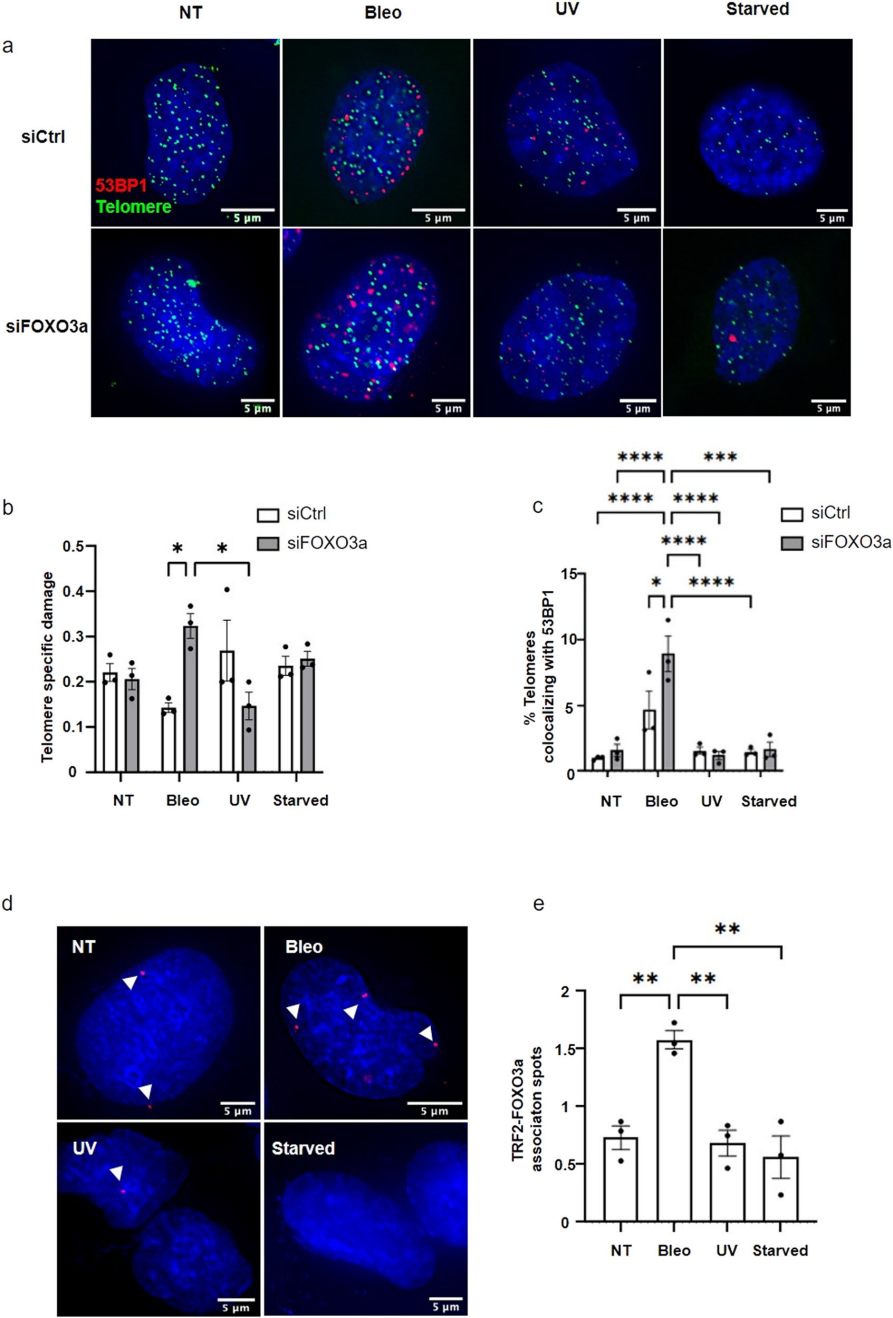
FOXO3a protects telomeres in BJ-HELT exposed to genotoxic stress. **a** TIFs in non-treated BJ-HELT cells or exposed to bleomycin, UV or starvation and downregulated or not for FOXO3a. Immunofluorescence of 53BP1 (red) and telomeric PNA-FISH probe (green). **b** Telomeric DNA specific damage quantification of representative images in (**a**). Statistical analyses were performed using one-way ANOVA test with Tukey’s post hoc test (*p < 0.05), in biological triplicates. **c** Percentage of damaged telomeres (colocalizing with 53BP1). Conditions were compared by a one-way ANOVA, in biological triplicates. **d** PLA showing TRF2-FOXO3a association in non-treated BJ-HELT or exposed to bleomycin (50 μg/mL, 24 hr), UV (UVA and UVB, 300 mJ/cm^2^) or under caloric restriction. PLA red foci are pointed by white arrows. **e** PLA images analysis, compared by a one-way ANOVA with Tukey’s post hoc test, *n* = 3 (**p < 0.01). Mean ± SEM are represented.

To identify the type of telomere damage that FOXO3a protects against, we exposed BJ-HELT cells to three types of stressors (bleomycin: bleo, starvation, and ultraviolet radiation: UV). Starvation is known to reduce cell proliferation^42^. UV triggers pyrimidine dimers and single strand DNA breaks, whereas bleomycin induces single and double DNA strand breaks, as well as base damage^43,44^. Only bleomycin-treated cells showed that *FOXO3a* downregulation increased the rate of sTIFs (Fig. 3a–c). Reminiscent of *TERF2* downregulation, FOXO3a specifically protected against ATM-recognized telomere damage in bleomycin-treated BJ-HELT cells (Supplementary Fig. 5a). Since both bleomycin and starvation settings increased the translocation of FOXO3a in the nucleus (Supplementary Fig. 5b, c), one cannot solely attribute the telomere protective properties of FOXO3a in bleomycin-treated cells to its activation and translocation to the nucleus.

As in TRF2 KD cells, we detected FOXO3a-telomere associations in bleomycin-treated cells as revealed by PLA of TRF2 and FOXO3a (Fig. 3d, e; Supplementary Fig. 5d, e). Importantly, upon bleomycin treatment such associations cannot be merely attributed to the effect of global nuclear translocation of FOXO3a and random interactions with telomeres as, upon starvation, which causes similar massive nuclear localization of FOXO3a, we did not observe significant PLA signal (Fig. 3d, e).

Noteworthy, our setting of UV exposure led neither to DNA damage activation nor specific telomere damage nor FOXO3a translocation, precluding any conclusion on the protective effect of FOXO3a-dependent telomeres in UV-irradiated BJ-HELT cells.

In mammals there are four FOXO proteins: FOXO1, FOXO3, FOXO4, and FOXO6^45^ with redundant roles^46^. We tested whether the telomere-protective function of FOXO3a is shared by other FOXO proteins by knocking down *FOXO1, FOXO3*, and *FOXO4* in BJ-HELT cells treated with bleomycin (Supplementary Fig. 5f). The rate of sTIFs increased only in the si*FOXO3* condition, indicating that telomere protection is restricted to FOXO3a among these members of the FOXO family.

We conclude that FOXO3a exhibit specific properties that protect telomeres upon ATM-recognized injuries, like those conferred by TRF2 downregulation or bleomycin.

### The CR2C domain but not the forkhead domain of FOXO3a is required for telomere protection

**Fig. 4:**
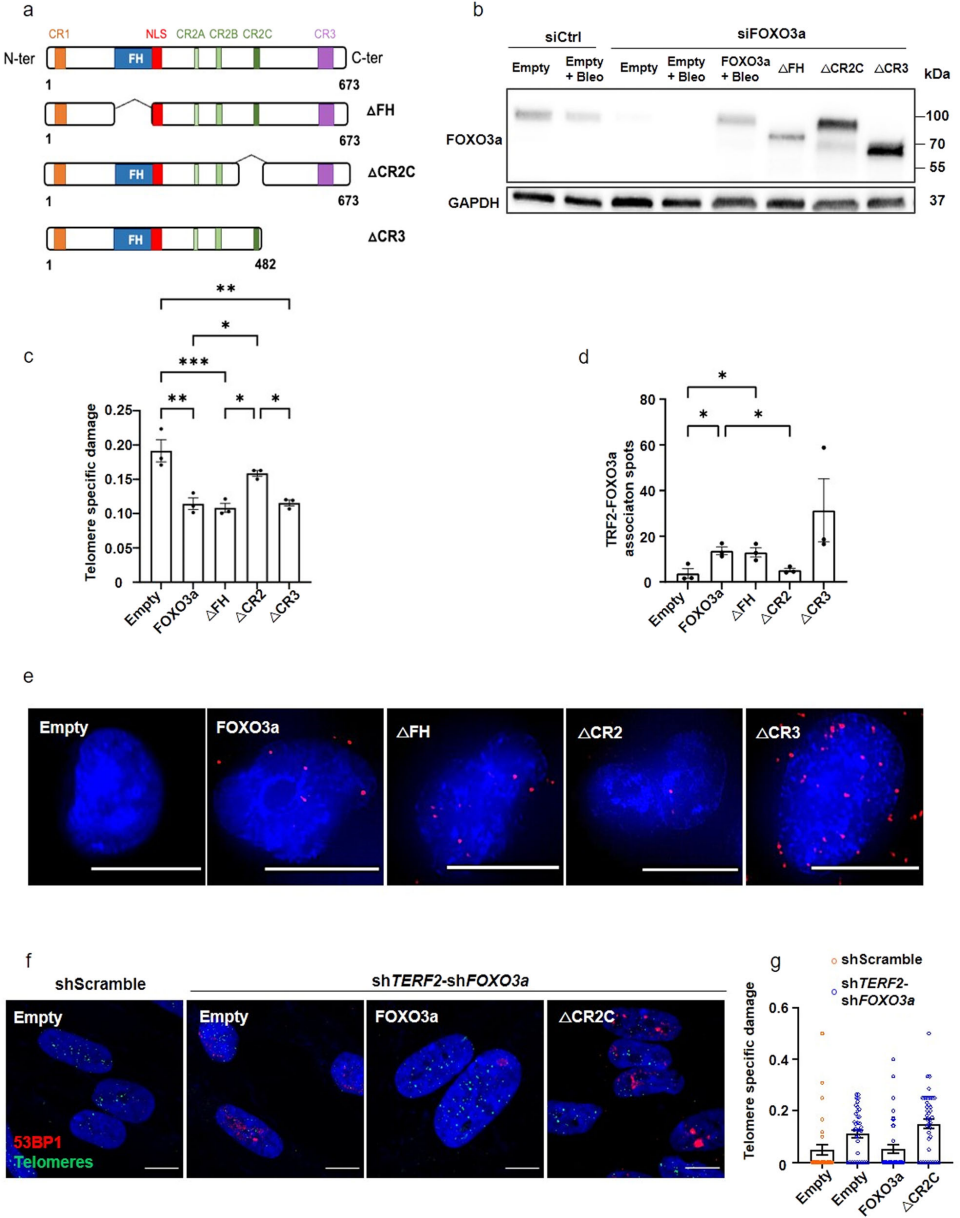
FOXO3a CR2C domain is required for telomere protection. **a** FOXO3a full-length structure (top) showing relevant domains and truncated mutant forms (ΔFH, ΔCR2C and ΔCR3). FOXO3a is composed by three conserved regions: CR1, CR2 and CR3 known to interact with several proteins. CR2 is divided in three subregions: CR2A, CR2B and CR2C. FOXO3a contains a Forkhead (FH) or DNA binding domain, that recognizes a TGTTTAC consensus DNA sequence, followed by a nuclear localization sequence (NLS). **b** Western blotting showing endogenous FOXO3a downregulation, FOXO3a full-length rescue and expression of FOXO3a truncated proteins. Cells were transduced 5 days with lentiviruses and the last 3 days transfected with siRNA. GAPDH was used as loading control. **c** sTIF quantification in FOXO3a KD BJ-HELT fibroblasts, overexpressing truncated of FOXO3a and treated with bleomycin, *n* = 3. Conditions were compared to siFOXO3a-FOXO3a+Bleo using a one-way ANOVA test (*p < 0.05, **p < 0.01, ***p < 0.001). **d** PLA showing TRF2-FOXO3a association in siFOXO3a + exogenous truncated FOXO3a overexpression in BJ-HELT treated with bleomycin, *n* = 3. Conditions were compared by a one-way ANOVA test, *p-value*: siFOXO3a-FOXO3a+Bleo *vs* siFOXO3a-Empty+Bleo: 0,0168; *vs* siFOXO3a-ΔCR2C + Bleo: 0,0368. **e** Representative images showing TRF2-FOXO3a association by PLA. Scale bar 10 μm. **f** Representative images of TIFs (telomeric probe in green and 53BP1 in red) in human myotubes downregulated for *TERF2* and *FOXO3a* and transduced with exogenous forms of FOXO3a. Scale bar 10 μm. Mean ± SEM are represented. **g** sTIF in human myotubes overexpressing FOXO3a constructions. The control condition, shScramble-Empty, is represented in white, whereas the sh*TERF2*+sh*FOXO3a* conditions are shown in gray. This graph shows quantification for a unique replicate, data of the second replicate are in Supplemental fig. 6.

Next, we investigated which domain of the FOXO3a protein conferred its specific properties of telomere protection. The FOXO3a protein domains include a forkhead DNA-binding domain (FH) and three conserved regions (CR1-3) with CR3 acting as the main transactivation domain. We constructed a series of lentiviral vectors expressing various truncated forms of FOXO3a: ΔFH, ΔCR2C, and ΔCR3 (Fig. 4a). By downregulating *FOXO3a* via RNA interference in cells overexpressing wild-type (WT) or truncated FOXO3a alleles, we obtained cells expressing almost physiological levels of FOXO3a or its truncated forms (Fig. 4b; Supplementary Fig. 6a). Importantly, expression of WT FOXO3a was sufficient to restore telomere protection in bleomycin-treated cells, indicating that the small interfering RNA pool used to downregulate *FOXO3a* expression does not cause off-target effects related to telomere protection (Fig. 4c). Only the form with deletion of the CR2C domain was unable to rescue telomere protection upon bleomycin treatment (Fig. 4c) and showed reduced FOXO3a telomere localization (Fig. 4d, e). We conclude that FOXO3a exerts a telomere-protective effect uncoupled from its canonical role as a DNA-binding transcription factor but dependent on the CR2C domain. Whether other FOXO3a domains than CR2C are involved in telomere protection will require further studies.

Finally, we asked whether the FOXO3a CR2C domain is also required for the telomere protective-function of FOXO3a in human myotubes with downregulated *TERF2* (Fig. 1). The expression of wild-type FOXO3a but not CR2C, rescued the telomere deprotection conferred by the double knockdown of *FOXO3a* and *TERF2* (Fig. 4f, g and Supplementary Fig. 6b–f). Hence, as in BJ-HELT cells, the CR2C domain of FOXO3a is required to protect telomeres in *TERF2*-disrupted human myotubes.

## Discussion

Mammalian FOXO transcription factors, homologs of DAF-16, which regulates longevity in *Caenorhabditis elegans*, play a key role in cellular homeostasis by upregulating genes associated with oxidative stress resistance, metabolism, apoptosis, cell cycle arrest, aging, and autophagy^7^. Here, we reveal a non-canonical function of FOXO3a in providing specific protection to telomeres against a genotoxic insult. Downregulation of *FOXO3a* in unchallenged cells has little or no effect on telomere protection, whereas FOXO3a is protecting telomeres in cells experiencing genotoxic stress. This conditional protective role of FOXO3a was observed in post-mitotic muscle fibers upon *TERF2* downregulation, leading to mitochondrial dysfunction and FOXO3a activation, as well as in transformed fibroblasts upon *TERF2* downregulation combined with treatment with the clastogenic agent bleomycin. These findings raise the question of the type of damage that causes FOXO3a to be efficiently recruited for telomere protection. Our results indicate that such damage is recognized via the ATM-dependent DDR pathway.

The telomere-protective function of FOXO3a cannot be simply explained by its general roles in DNA repair and oxidative stress^47–49^. First, by normalizing the rate of telomere damage to the total amount of DNA damage, telomeres appear to be better protected than the rest of the genome upon *FOXO3a* downregulation. Second, in *TERF2*-compromised myotubes, the total amount of ROS remained unchanged upon *FOXO3a* downregulation. Third, a subset of FOXO3a proteins is associated with telomeres in myotubes upon *TERF2* downregulation and in fibroblasts upon bleomycin treatment, as assayed by co-localization with telomeric PNA probe foci, ChIP and co-IP. Notably, this telomere association may be uncoupled from FOXO3a nuclear translocation. Indeed, starvation, which causes massive nuclear translocation of FOXO3a, was not correlated with an increase in telomere association; conversely, bleomycin treatment increased recruitment of FOXO3a at telomeres whereas the rate of FOXO3a translocation was lower than that caused by starvation. These findings suggest that FOXO3a undergoes post-translational modification in response to genotoxic stress to associate with telomeres.

The telomere association and protective roles of FOXO3a were conserved upon deletion of its FH DNA-binding domain or its C-terminal CR3 transactivation domain. These results rule out the possibility of FOXO3a acting at telomeres through its canonical role as a DNA-binding transcription factor. Moreover, deletion of CR3 increased the ability of FOXO3a to associate with telomeres. This finding suggests competition between the major transactivation domain of FOXO3a and its telomere-specific functions. Deletion of CR3 may also cause FOXO3a to exhibit an open conformation^50^ that is more prone to telomere association. The CR3 domain was reported to bind and activate ATM^51,52^, making a protective role for FOXO3a at telomeres via its interaction with ATM unlikely. In contrast to the FH and CR3 domains, deletion of CR2C impaired the telomere-protective effect of FOXO3a. CR2C exhibits transactivation activity and mediates the association of FOXO3a with SIRT1^53^ and with CBP/p300^54,55^. In accordance with involvement of the FOXO3a–p300 complex in telomere protection, p300, but not CBP, was associated with telomeres^56^, suggesting that acetylation mediated by p300 promotes the repair of damaged telomeres. This model requires substantiation, particularly by identifying the targets of FOXO3a–p300 on telomeres. One candidate is TRF2, which contains a p300 binding site^57^. Whereas FOXO3a have been closely related to longevity in several species, as studied in deletion and point mutation, any animal model carrying a mutation in the CR2C domain has been created or characterized yet. Moreover, SNP associated to extreme longevity in humans are not carried in the CR2C sequence. FOXO3a^-/-^ mice are viable but females develop ovarian problems during early adulthood^58^. We could imagine FOXO3a ^ΔCR2C^ mice might have an increased sensitivity to genotoxic insults and a premature sarcopenic phenotype.

The fact that an oxidative stress in skeletal muscle cells has a positive effect is not unprecedented. For instance, muscular exercise promotes health benefits but also ROS production^59^, which may mediate adaptive responses by facilitating glucose uptake or inducing mitochondrial biogenesis^60,61^. Our results extend the beneficial effect of ROS signaling to telomere protection via FOXO3a activation. This mechanism can be viewed as a safeguard pathway of telomere stability upon oxidative stress in response to muscular exercise and physiological demand. In this respect, it is interesting that *TERF2* expression is downregulated during high intensity training^62^, suggesting that the TRF2-independent mechanism of telomere protection conferred by FOXO3a, revealed here, is important in muscle adaptation to exercise.

Overall, our findings establish a direct link between two key longevity pathways, FOXO3a^45^ and telomere protection^63–65^. Importantly, our results indicate that this connection is involved in muscle homeostasis and aging. Indeed, telomere protection relies on FOXO3a in *TERF2*-depleted muscle fibers, which occurs during muscle development in young adults and is exacerbated during aging^21^. Furthermore, the FOXO3a–telomere increased association with age. We propose that telomeres of skeletal muscle cells are protected in a privileged manner by FOXO3a during aging, supporting a synergized action between telomere and FOXO3a to regulate longevity.

## Methods

### Cell culture

Human myoblasts were isolated from patient 12 (Supplementary Fig. 3) and immortalized^21^. For cell culture maintenance, cells were platted in dishes coated with 0.1% pigskin gelatin in 4:1 Dulbecco modified Eagle medium/Medium 199 supplemented with 15% FBS, 0.02 M HEPES, 1.4 mg/l vitamin B12, 0.03 mg/l ZnSO_4_, 0.055 mg/l dexamethasone, 2.5 μg/l hepatocyte growth factor and 10 μg/l basic fibroblast growth factor. Cultures were passaged at ~70% confluency. Differentiation into myotubes was started following a change to differentiation medium (2% horse serum in 4:1 Dulbecco modified Eagle medium: Medium 199) when 90% confluent.

For infection of myotubes, myoblasts were seeded in 6 cm or 10 cm dishes, switch to differentiation media (2% Horse Serum) upon confluence (90%) and transduced at least week after. Cells were transduced at a MOI 2 using the different shRNAs and MOI 4 for the overexpression vectors.

BJ-HELT cells were immortalized by transduction of *hTERT* and *SV40* ER genes^66^. BJ-HELT cells were grown in DMEM supplemented with 10% fetal bovine serum, penicillin (100 IU/ml), and streptomycin (100 μg/ml). Cells were cultured at 37 °C, 5% CO_2_.

### Lentivirus infection and siRNA transfection

Lentiviruses were produced by transient calcium phosphate transfection of HEK293T cells with the virus packaging plasmids, p8.91 and pVSVg and a pWPIR-GFP lentiviral expression vector or pLKO plasmid, that contained the sequence of interest. We used MISSION shRNAs (pLKO.1; Sigma): *TERF2* (TRNC0000004812), *FOXO3A* (TRNC0000010335, only clone validated by Sigma MISSION shRNAs, to date) and control (SHC002). For upregulation, the pWPIR-GFP backbone was used and modified by Genscript to reach full-length FOXO3a pWPIR-FOXO3aWT-GFP and mutant forms pWPIR-ΔFH-GFP, pWPIR-ΔCR2C-GFP, pWPIR-ΔCR3-GFP. pWPIR-TRF2-GFP and pWPIR-TRF2ΔB-GFP vectors have already been validated^32^. Transduction efficiency was determined by one-week puromycin treatment and count the number of resistant clones for pLKO.1 vectors and GFP-flow cytometry detection after 3 days for pWPIR-GFP vectors.

siRNA transfections were performed with On-Target Plus SMARTpool (Dharmacon) and Dharmafect1 transfection reagent (T-2001, Dharmacon) for 72 hr.

Efficiency of each shRNA and siRNA was checked routinely by RT–qPCR or Western blotting.

### Antioxidant treatment

Myoblasts undergoing differentiation were treated with 5 mM N-acetylcysteine (NAC: A9165 Sigma) and 10 mM epigallocatechin gallate (EGCG: E4143 Sigma) once myoblast fusion process was over. At this same time point, cells were transduced with shRNA and overexpressing vectors. Myotubes were incubated with the antioxidant solutions for 7 days. Media was changed every 2 days to refresh the antioxidant content.

### Cellular stressors treatments

BJ-HELT cells were treated with Bleomycin (Merk B2434), resuspended in distilled water and added to cell media at 50 μg/mL for 24 h. For UV irradiation, media was removed, cells were washed with PBS and kept in a minimal volume of PBS. Then, cells were irradiated with UVA and UVB at 300 mJ/cm^2^ and let to recover for 2 hr before collection or fixation. To induce starvation, BJ-HELT cells were grown in normal conditions up to 70% confluency. Then, FBS containing media was removed, cells were washed with DMEM once and add DMEM without FBS. Cells were incubated under starvation for 1 week.

### Human biopsies and Ethic statement

The collection of fetal muscle biopsies was approved by the “Agence Française de la Biomedecine” of the Ministery of Health for legal access to the biological material in full accordance with the law (research protocol number PFS13-006). Samples were obtained after therapeutic abortion. Parents have provided written informed consent for the use of biopsies for medical research in accordance with the Declaration of Helsinki. Muscle biopsies were processed by fetopathologists from fetuses not affected by a muscular pathology. Skeletal muscle biopsies from teens and adults were obtained from the Nice Hospital (CHU l’Archet registered as protocol number DC-2015 2374) and from the Tumorothèque, Assistance Publique des Hôpitaux de Marseille, agreement n°AC-2013-1786, from healthy donors using standardized muscle biopsy protocol.

### Immunofluorescence-FISH and Telomere damage Induced Foci (TIFs)

Cells were grown onto glass coverslips or multichamber slides and fixed for 20 min with 3.7% formaldehyde. Cells were then permeabilized with 0.5% Triton X-100 for 15 min and dehydrated in increasing concentrations of ethanol for 3 min (50%, 70% and 100%). Hybridization of PNA probes was performed for at least 2 h at RT after denaturation (5 min) in 70% formamide, 10 mM Tris pH 7.2 and 1% blocking solution (Roche) at 80 °C. Then, cells were washed in a 70% formamide, 10 mM Tris pH 7.2 solution for 30 min, followed by washes with 150 mM NaCl and 50 mM Tris pH 7.5 for 15 min. Next, the cells were incubated 1 h with blocking buffer (3% BSA and 0,3% Triton X-100) and incubated overnight at 4 °C with the desired antibody: FOXO3a, rabbit monoclonal (clone 75D8, 1:200, Cell Signaling Technology) and 53BP1, rabbit polyclonal (NB 100-305, 1:200, Novus Biologicals). Cells were then washed with 1X PBS and incubated for 1 h with the corresponding secondary antibody. Finally, slides were prepared with a DAPI containing mounting solution (Vectashield, Vector Laboratories).

Pictures were taken using a DeltaVision Elite system (GE). An average of 70 stacks and 40 nuclei were taken per conditions. Images were then analyzed using FIJI (ImageJ). Total number of 53BP1 foci was determine and telomere-53BP1 colocalizations (TIFs) were determined by doing a stack-by-stack screening looking for at least one colocalizing pixel (yellow).

The telomeric specific damage was calculated as the ratio between the number of TIFs and the total number of 53BP1 foci.

### Immunofluorescence

Cells were grown onto glass coverslips or multichamber slides and fixed for 20 min with 3.7% formaldehyde. Cells were then permeabilized with 0.5% Triton X-100 for 15 min and blocked for 1 h with (3% BSA and 0,3% Triton X-100). Next, cells incubated overnight at 4 °C with the desired antibody: FOXO3a, rabbit monoclonal (clone 75D8, 1:200, Cell Signaling Technology). Cells were then washed with 1X PBS and incubated for 1 hr with the corresponding secondary antibody. Finally, slides were prepared with a DAPI containing mounting solution (Vectashield, Vector Laboratories H-1200).

### Reactive Oxygen Species (ROS)

Cells were grown on cover slides and treated as indicated in the manufacturer’s ROS kit instructions (Enzo-51011). This kit recognizes different reactive species (hydroxyl radicals, hydrogen peroxide, peroxynitrite) but does not detect superoxide. Transduced differentiated cells were washed twice and 1 ml of fresh differentiation media was added, with or without EGCG (10 mM) or H_2_O_2_ (100 μM) and incubated for 30 min at 37 °C. After additional wash and media renewal (1 ml), cells were incubated for 1 h at 37 °C with a solution composed 2X ROS detection and Oxidative stress reagent (5 mM; dilution 1:2500). Cells were then washed with 1X PBS and directly mounted using 15 μl of vectashield+DAPI, without any fixation. Pictures were taken using a DeltaVision Elite system (GE). An average of 100 stacks and 50 nuclei were taken per conditions. Images were then treated using IMARIS. Intensities of ROS foci and DAPI staining were used for analyses, excluding single-nuclei cells for myotubes analysis.

### Proximity Ligation Assay (PLA)

Cells were, fixed with 3,7% formaldehyde, permeabilized with 0.5% Triton X-100 for 15 min, and blocked with Duolink kit Blocking solution (DUO94001, Sigma-Aldrich). Cells were then incubated with primary antibodies to TRF2, mouse monoclonal, (NB100-56506, 1:300, Novus Biologicals), FOXO3a, rabbit monoclonal (clone 75D8, 1:1500, Cell Signaling Technology). Then, cells were incubated for 1 h at 37 °C with DNA-linked secondary antibodies (PLA probes), analyzed with the Duolink In Situ Red Mouse/Rabbit kit assay according to the manufacturer’s instructions (DUO94001, Sigma-Aldrich). In brief, the ligation and amplifications steps were performed for 30 min and 2 hr, respectively, at 37 °C. Finally, cells were fixed again with formaldehyde 37 °C. Images were taken with Deltavision Elite® and analyzed with FIJI software. Only nuclear spots were considered for analysis. Each antibody used was first tested by immunofluorescence. PLA negative controls were performed to test each couple of antibodies: each antibody alone or without any antibody but with mouse/rabbit PLA probes.

### Chromatin-Immunoprecipitation (ChIP)

Samples were crosslinked for 10 min at RT and 20 min at 4 °C with 0.8% formaldehyde (methanol free, ultrapure EM grade, Polysciences, Inc; Warrington PA). Reaction was stop at RT for 10 min with the addition of Glycine to a final concentration of 0.125 M. Cells were rinsed twice with ice-cold 1X PBS, scraped from the dish and pelleted after centrifugation (800 g, 5 min at 4 °C). For sonication, we used a total processing time of 15 min per sample in a Bioruptor (Diagenode) using the following settings: 14 cycles; 30 s ON/30 s OFF on High power. Sonicated DNA was controlled on a 2% agarose gel, adequate sonication is achieved when a smear ranging from 200-700 bp is obtained. IPs were processed using a 4 °C O/N incubation with TRF2 antibody at 1.5 μg; (Novus: NB100-56506); 1 μl of each preparation: IP, IgG, Rabbit non-immune Serum. Next, magnetic beads (Dynabeads, Life Technologies) were added for 3 h. Samples were washed with a low salt buffer (150 mM NaCl, 1% Triton X-100, 0.1% SDS), a high salt buffer (500 mM NaCl, 1% Triton X-100, 0.1% SDS) and a lithium salt buffer (0.25 M LiCl, 1% NP40, 1% deoxycholic acid). Chromatin was eluted (1% SDS, 0.1 M NaHCO_3_ solution), and the cross-linked chromatin was reversed at 65 °C overnight. The DNA was treated with RNaseA and proteinase K, followed by phenol–chloroform purification. The DNA obtained from ChIP was denatured and blotted onto nylon membranes using a slot blot apparatus, cross-linked, and hybridized with telomere and Alu repeats radioactively labeled probes. The membranes were exposed onto phosphorimager screens, and the signal intensity was quantified with ImageQuant software.

### Co-Immunoprecipitation

Skeletal muscle human biopsies were lysed in 50 mM Tris-HCl, 150 mM NaCl and 0,1% NP40; complemented with phosphatase inhibitors (Roche). Samples were sonicated using a Bioruptor (Diagenode) for 5 min (30 s ON/ 30 sOFF on High Power). Then, tubes were centrifugated 5 min at 4 °C, 12,000 g. 10% of each sample was used for Input. Immunoprecipitations were processed using a TRF2 antibody (Novus: NB100-56506) at 4 °C, O/N incubation. Next, magnetic beads (Dynabeads Protein G, Thermofisher, 10004D) were added for 1 h at 4 °C. Samples were washed with 50 mM Tris-HCl, 150 mM NaCl and 0,1% NP40; complemented with phosphatase inhibitors (Roche), five times. Resuspend samples in 2X Laemmli. Finally, samples were denaturated at 95 °C for min and load then on a SDS-PAGE gel for Western Blot running.

### Western Blot

Cells were collected in 1X PBS and spin down (250 g, 5 min) and pellet stored at -80 °C for further use. Whole cell lysates were prepared from cells by adding cell lysis RIPA buffer, complemented with phosphatase inhibitors (Roche) and kept 30 min on ice. For each sample, 30 μg of protein was resolved in a 4-15% gradient mini-protean precast polyacrylamide gels (BioRad) and transferred to nitrocellulose membranes (Whatman, GE Healthcare) for 1 h at 4 °C. After blocking for 1 h with 5% skim milk in PBST (0.1% Tween-20 in PBS), the membranes were incubated overnight at 4 °C with primary antibodies diluted in 5% milk. The following primary antibodies were used: TRF2, mouse monoclonal, (NB100-56506, 1:1000, Novus Biologicals); TRF2, rabbit monoclonal, (NB110-57130, 1:5000, Novus Biologicals); FOXO3a, rabbit monoclonal (clone 75D8, 1:1500, Cell Signaling Technology); GAPDH, rabbit polyclonal (1:2000, NB100-56875, Novus Biologicals). The membranes were then rinsed three times in PBST for 10 min and incubated 1 h at room temperature with appropriate secondary antibodies diluted (1:5000) in 5% PBST-milk (e.g. anti-mouse HRP IgG and anti-rabbit HRP IgG, Vector Labotratories). Membranes were developed using the Luminata Forte HRP substrate (Millipore) and exposed in the Fusion Solo apparatus (Vilbert Lourmat).

### RT-qPCR

Myotubes RNA was extracted by Tri Reagent (Trizol; Sigma T9424), following manufacturer’s instructions. BJ-HELT and myoblasts were lysed (RNeasy plus kit (Qiagen: 74034)). Total RNA purified according to the manufacturer’s instructions and was quantified on a Nanodrop 1000 spectrophotometer (Thermo Scientific). For Reverse Transcription (RT) 500 ng RNA was reverse transcribed High-Capacity RNA-to-cDNA Kit (Thermo Scientific). Each qPCR contained 5X diluted cDNA, 0.2 μM primers, and SYBR Green Master Mix (Roche, 4913914 001). Quantitative RT-PCR (qRT-PCR) was permorfed in triplicates using FastStart universal SYBR Green master Mix (Roche). Melting curves were analyzed (SYBR green) to exclude non-specific amplification products. We confirmed amplicon size at least once on agarose gels. Crossing-threshold (Ct) values were normalized by subtracting the geometric mean of two housekeeping genes (GAPDH and HPRT).

### Statistics and reproducibility

Statistical analysis was performed using the Prism 9 software (GraphPad). Quantitative data are displayed as means ± standard error of the mean. For comparison of two groups, we used two-tailed Mann–Whitney U-test or Student’s t-test. For comparisons of more than two groups, we used one-way ANOVA with Tukey’s post hoc test. *p-value* < 0.05 were considered significant (*p < 0.05, **p < 0.001, ***p < 0.001, ****p < 0.0001).

### Reporting summary

Further information on research design is available in the Nature Portfolio Reporting Summary linked to this article.

## Supplementary information

Gilson_Peer Review File

Supplementary Information

Description of Additional Supplementary Files

Supplementary Data 1

Reporting Summary

## Data Availability

All data are available in the main text or the supplementary materials. Source data are provided in Supplementary Data 1. Uncropped blots are provided in Supplementary Figs. 7–12.
