# Supplementary material for: Non-canonical telomere protection role of FOXO3a of human skeletal muscle cells regulated by the TRF2-redox axis
Source: Commun Biol. 2023 May 25;6:561. doi: 10.1038/s42003-023-04903-1 (PMC10213016; doi:10.1038/s42003-023-04903-1)

Supplemental Figures

Supplemental Figure 1

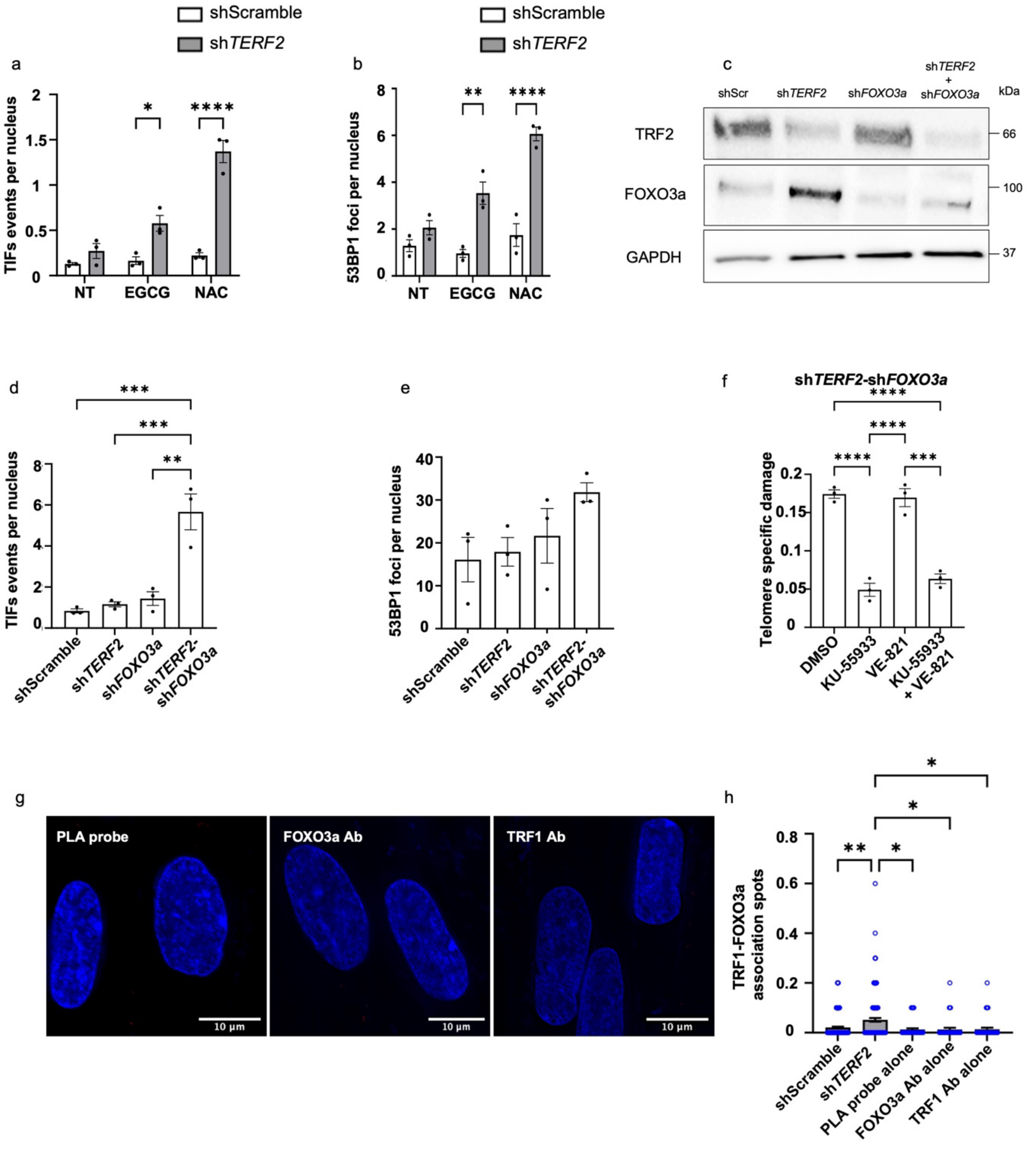

Supp. Figure 1: FOXO3a protects telomeres in *TERF2*-compromised human myotubes

a TIFs quantification: number of colocalization between the telomeric probe and 53BP1 foci, in myotubes downregulated for *TERF2* and treated with antioxidants (EGCG and NAC). n=3. Statistical analyses were performed using a two-way ANOVA assay. **b** Total number of 53BP1 foci. 30-40 nuclei were analyzed per replicate and per condition, n=3. Error bars represent SEM of three biological replicates, $*p < 0.05$, $**p < 0.01$, $***p < 0.001$ **c** Western Blotting showing *TERF2* and *FOXO3a* downregulation. In *shTERF2* myotubes, FOXO3a expression is increased. **d** and **e** TIFs and 53BP1 quantification in myotubes + sh*TERF2* and/or sh*FOXO3a*. Conditions were compared by a one-way ANOVA test, n=3, $**p < 0.001$, $***p < 0.001$. Means ± SEM are shown. **f** Telomeric specific damage (TIFs/53BP1 ratio) in myotubes inhibited for ATM and/ or ATR. 5 days after differentiation and 5 days before harvesting, ATM or ATR was inhibited using either KU-55933 (10mM) or VE-821 (10mM), respectively. Fresh media with chemical inhibitors was added every 2 days. Statistics determined by a one-way ANOVA assay, $***p < 0.001$, $****p < 0.0001$, n=3. **g** Representative images of Proximity Ligation Assay (PLA) TRF1-FOXO3a negative control in myotubes. Three different negative controls were carried out: each primary antibody was incubated alone or PLA probe was incubated alone. Quantifications are showed in **h**, Kruskal-Wallis, error bars indicate SEM, $p < 0.05$, $**p < 0.001$.

Supplemental Figure 2

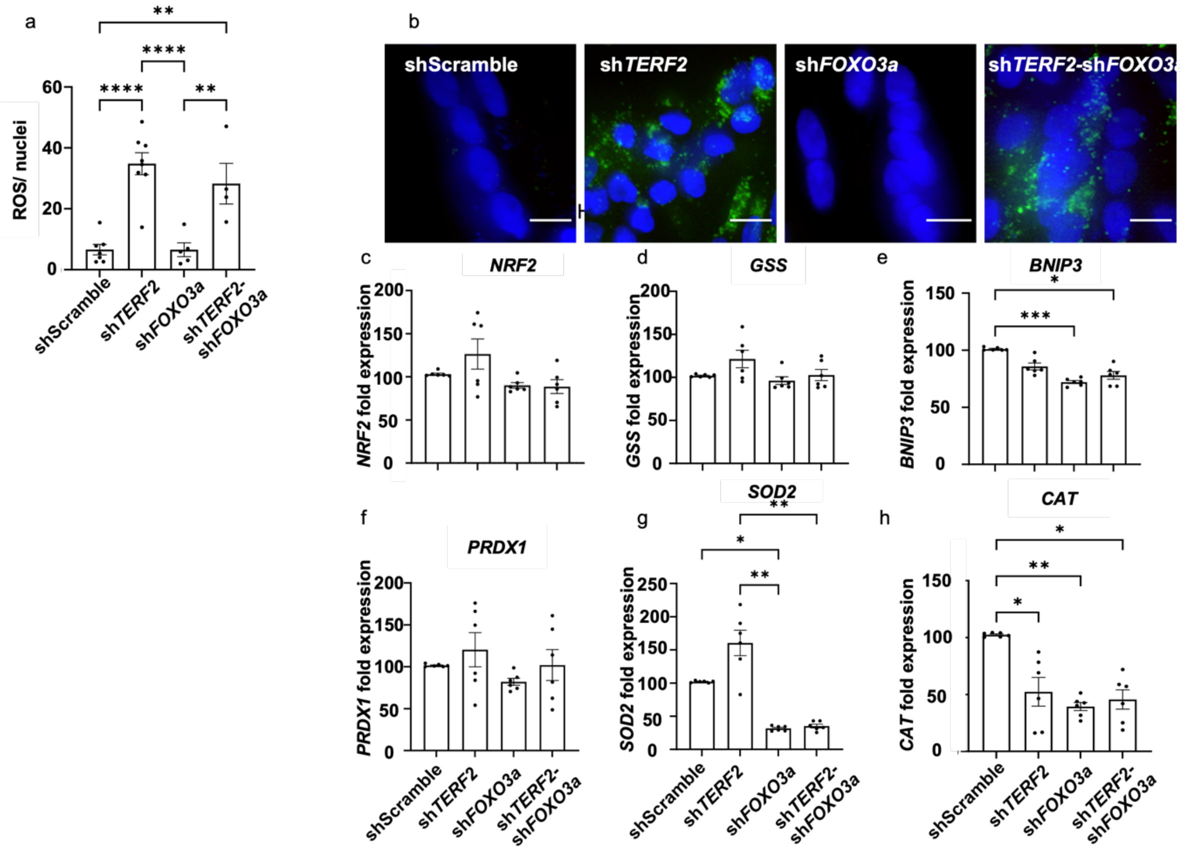

Supp. Figure 2: Telomere damage is not due to FOXO3a downregulation induced oxidative stress.

a Total number of ROS foci normalized to the number of nuclei. Downregulation of *TERF2* significantly increases ROS. Statistical analyses were performed using one-way ANOVA test (**$p < 0.001$, ****$p < 0.0001$). **b** Representative images of ROS foci in transduced myotubes. Scale bar 20 μm. **c** to **h** Gene expression quantified by RT-qPCR in transduced myotubes. Each measure represents the average fold-change expression of six independent repetitions, normalized to two housekeeping genes (HKG: HPRT and GAPDH); Pfaffl method). Statistical analyses were performed using the Kruskal–Wallis test (*$p < 0.05$, **$p < 0.001$, ***$p < 0.001$). Means ± SEM are shown.

Supplemental Figure 3

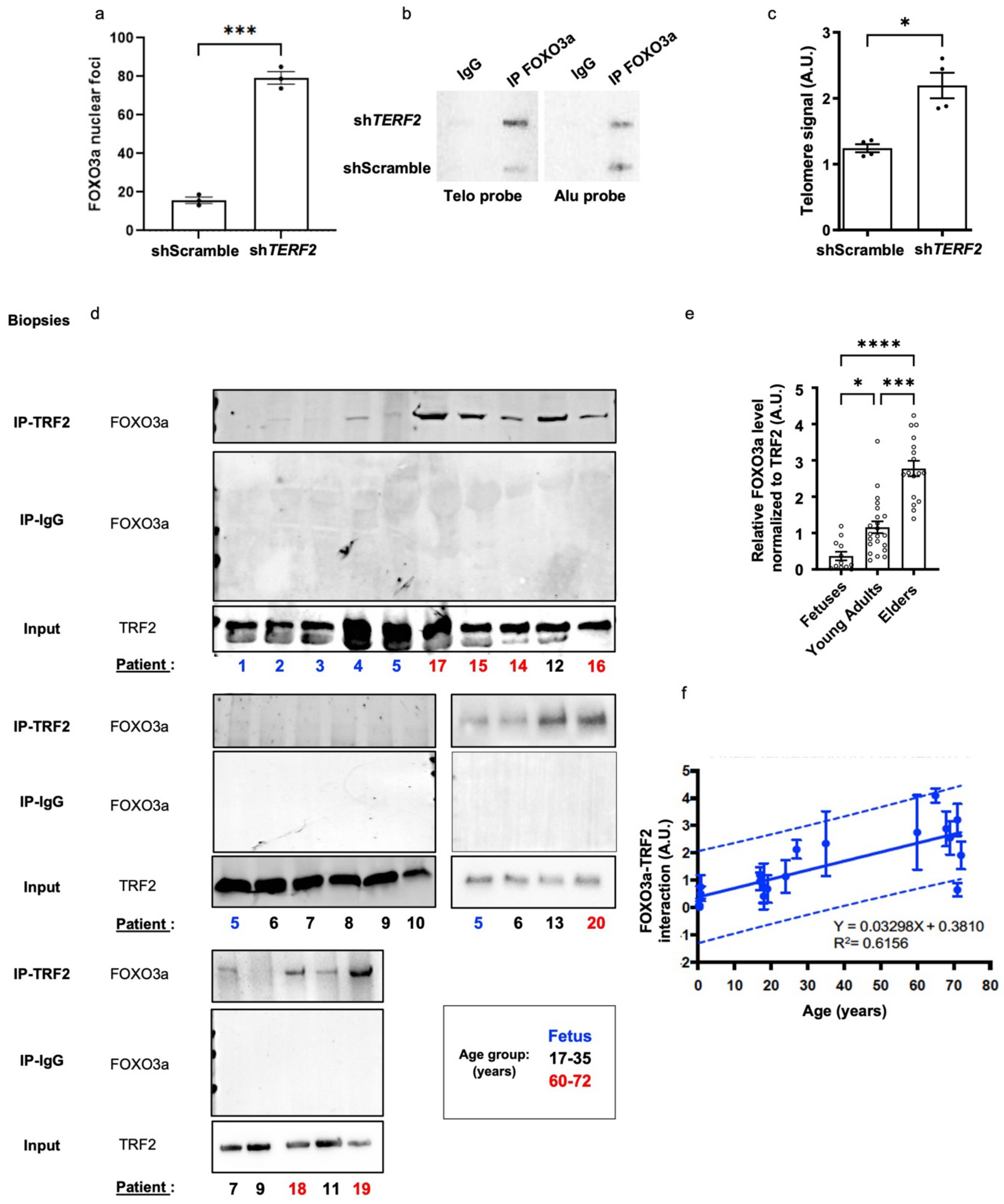

Supp. Figure 3: FOXO3a binds to telomeric DNA in myotubes downregulated for *TERF2*

a Quantification of nuclear FOXO3 foci in myotubes. An unpaired t-test was used for statistical analyses, n=3, ***$p < 0.001$. Means ± SEM are shown. **b** Slot blot of chromatin immunoprecipiated (ChIP) samples using a Telomeric (right) and Alu (left) probe in transduced myotubes. **c** ChIP associated quantification, FOXO3a telomeric signal was normalized to Alu repeats. n=4, statistical analyses were performed using an unpaired t-test. **d** to **f** Immunoprecipitation (IP) using TRF2 antibody in human skeletal muscle biopsies, using TRF2 antibody for precipitation and FOXO3A for revelation. The TRF2-FOXO3A interaction enhances with ageing. Statistical analyses were performed using the Kruskal–Wallis test (*$p < 0.05$, ***$p < 0.001$, ****$p < 0.0001$). **f** Correlation and goodness of fit associated (R^2), 95% interval confidence (blue lines) and means ± SEM are shown.

Supplemental Figure 4

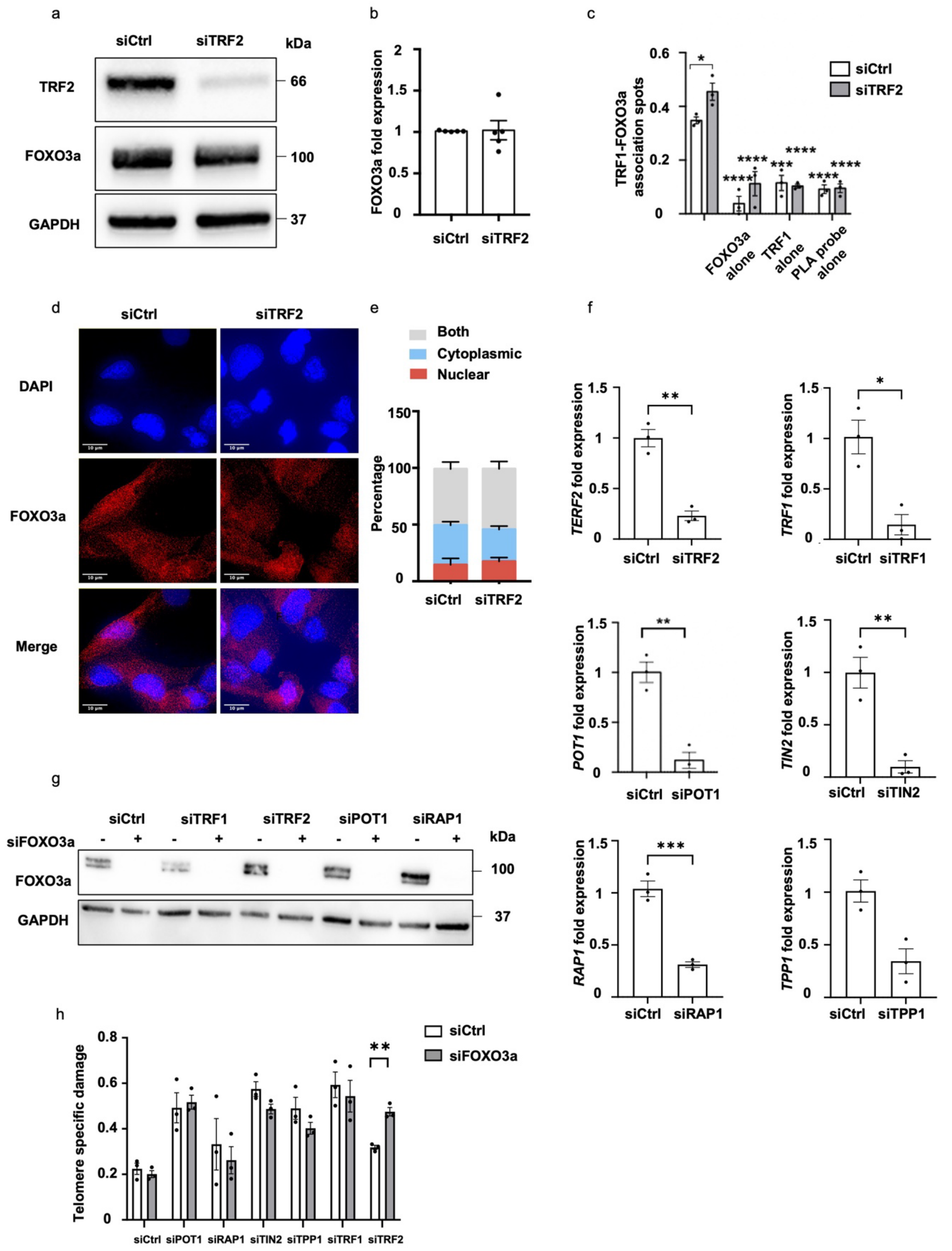

Supp. Figure 4: FOXO3a protects telomeres specifically upon *TERF2* downregulation in BJ-HELT cells.

a Western Blotting showing TRF2 and FOXO3a protein levels in TRF2 compromised BJ-HELT cells. **b** FOXO3a expression observed by WB upon TRF2 downregulation. Data represent the mean ± SEM of six biological replicates. Statistical analyses were performed using an unpaired t-test. **c** PLA TRF1-FOXO3a negative controls in BJ-HELT cells. Three negative controls were performed: incubation with each primary antibody alone or with the PLA probe alone. A two-way ANOVA, followed by a Bonferroni test was used for statistical analyses (*$p < 0.05$, ***$p < 0.001$, ****$p < 0.0001$). **d** and **e** FOXO3a immunofluorescence and qualitatively determination of FOXO3a cellular localization: mostly nuclear, mostly cytoplasmic, nuclear and cytoplasmic. Approximatively 60 cells were analyzed per condition in biological triplicates. **f** Shelterin proteins expression, measured by qPCR to validate siRNA downregulation efficiency. An unpaired t-test was used for statistical analyses, n=3, (*$p < 0.05$, **$p < 0.01$, ***$p < 0.001$. Means ± SEM are shown. **g** Western Blotting showing FOXO3a protein levels in BJ-HELT cells downregulated for each shelterin protein. **h** Telomere specific damage in BJ-HELT fibroblasts transfected with siRNA against each shelterin protein (TRF1, TRF2, POT1, RAP1, TIN2 and TPP1) individually (white) or combined to siFOXO3a (grey). Data represent the mean ± SEM, n=3, unpaired multiple t-test, **$p < 0.01$.

Supplemental Figure 5

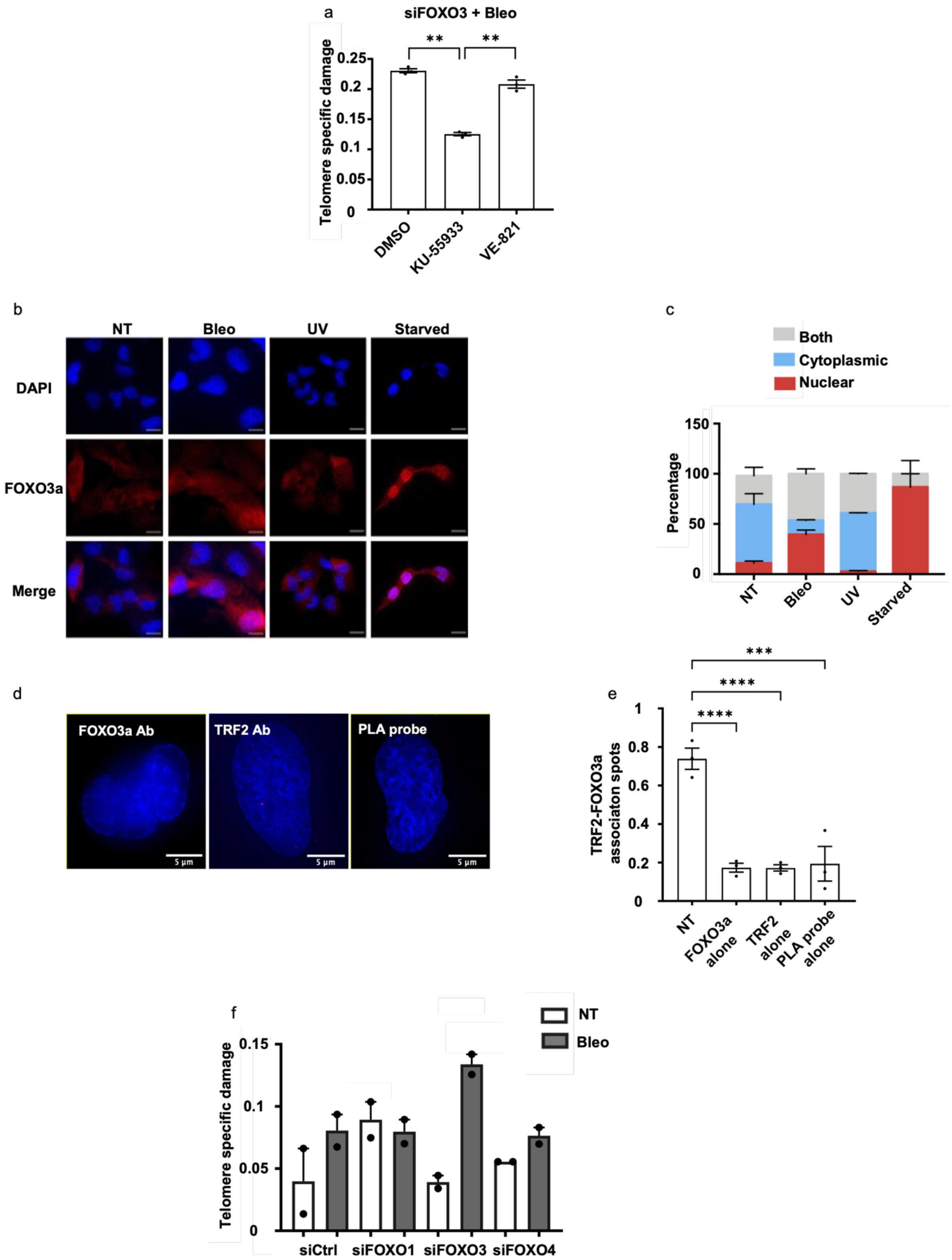

Supp. Figure 5: FOXO3a cellular localization is not affected by bleomycin in BJ-HELT fibroblasts

a Specific telomere damage in siFOXO3a-BJ-HELT cells, treated with bleomycin (50 μg/mL, 24hr) and inhibited for ATM (KU-55933) or ATR (VE-821). Three biological replicates analyzed with a one-way ANOVA test, $^{**}p < 0.01$. **b** FOXO3a cellular localization upon different kind of stress. Scale bar 10 μm. FOXO3a pattern was qualitatively determined as mostly nuclear, mostly cytoplasmic, nuclear and cytoplasmic **in (c).** Approximatively 30 cells were analyzed per condition in biological triplicates. **d** Representative images of PLA negative controls: FOXO3a antibody alone + PLA probe, TRF2 antibody alone + PLA probe, no antibodies but PLA probe alone. **e** PLA negative control quantification, compared to PLA (TRF2, FOXO3a antibodies and PLA probes added). Kruskal-Wallis' multiple comparisons test ($^{***}p < 0.001$; $^{****}p < 0.0001$) in three biological replicates. **f** Telomere specific damage in BJ-HELT cells downregulated for FOXO1, FOXO3 and FOXO4 and exposed to bleomycin treatment. Two biological replicates, 30-40 cells per condition. Data represents Mean ± SEM.

Supplemental Figure 6

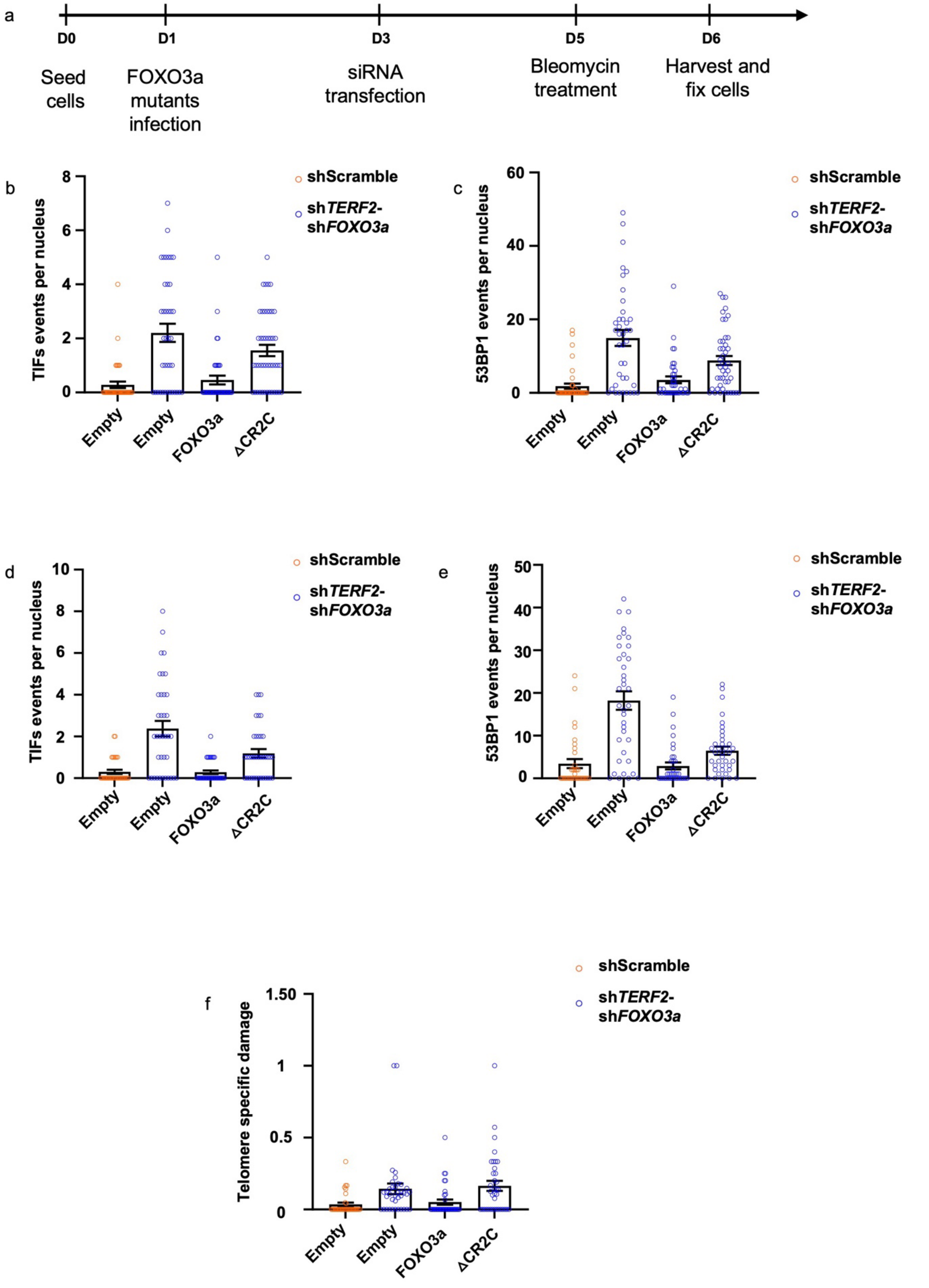

Supp. Figure 6: FOXO3a lacking domain mutant assay.

a FOXO3a truncated mutant overexpression and endogenous FOXO3a downregulation technical strategy. Cells were platted at day 0 and transduced at MOI 2 with lentiviral FOXO3a full-length or truncated forms and Empty vector at day 1. At day 3, each condition was transfected with siControl or siFOXO3a. At day 5, bleomycin (50 μg/mL) was added and cells were fixed or harvest 24hr later. **b** TIFs and **c** 53BP1 in human myotubes downregulated for endogenous FOXO3a overexpressing FOXO3a constructions. Results show mean ± SEM of the first biological replicate. results from the biological replicate. **d** TIFs, **e** total 53BP1 and **f** sTIFs in human myotubes downregulated for endogenous FOXO3a overexpressing FOXO3a constructions. Data is represented in mean ± SEM, and correspond to the second biological replicate.

Supplemental Figure 7

Uncropped blot Fig. 4b

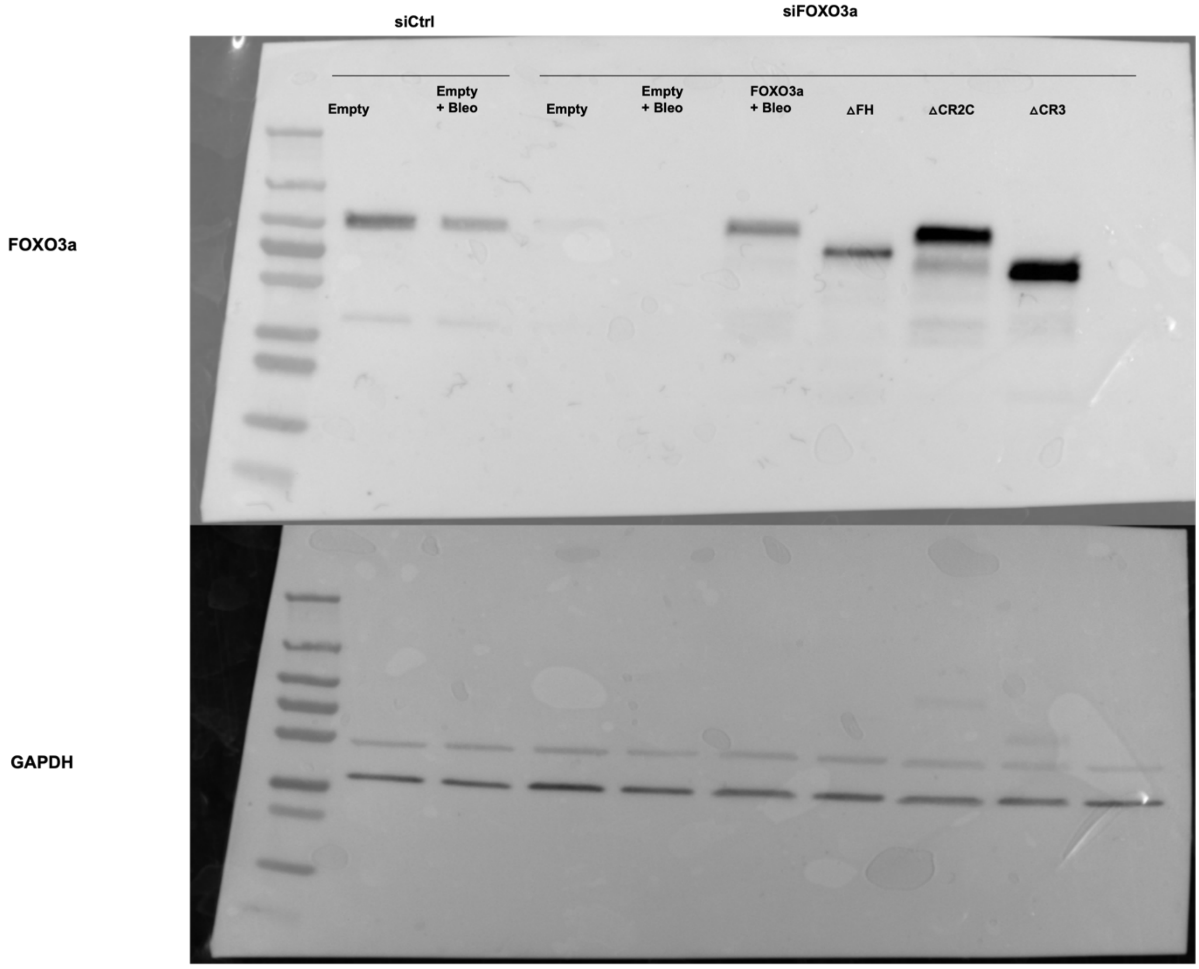

Supplemental Figure 8

Uncropped blot Supplemental Fig. 1c

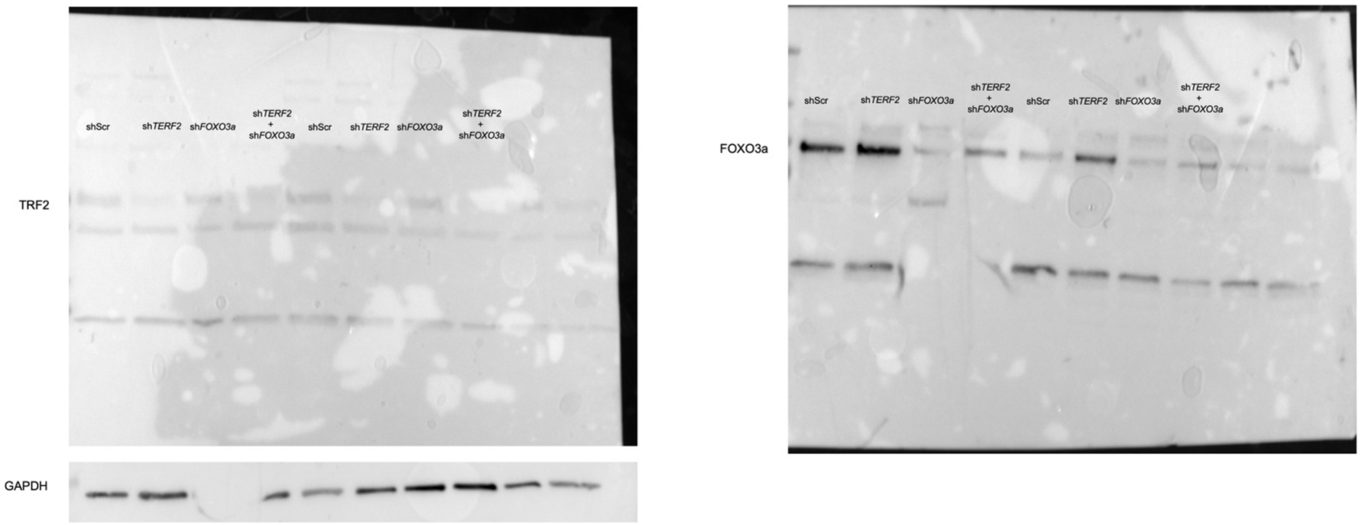

Supplemental Figure 9

Uncropped blot Supplemental Fig. 3b

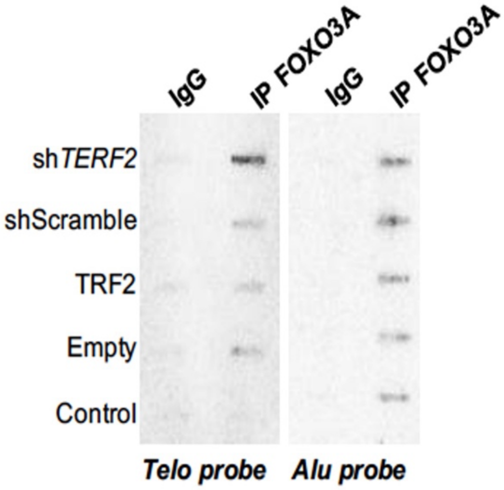

Supplemental Figure 10

Uncropped blot Supplemental Fig. 3d

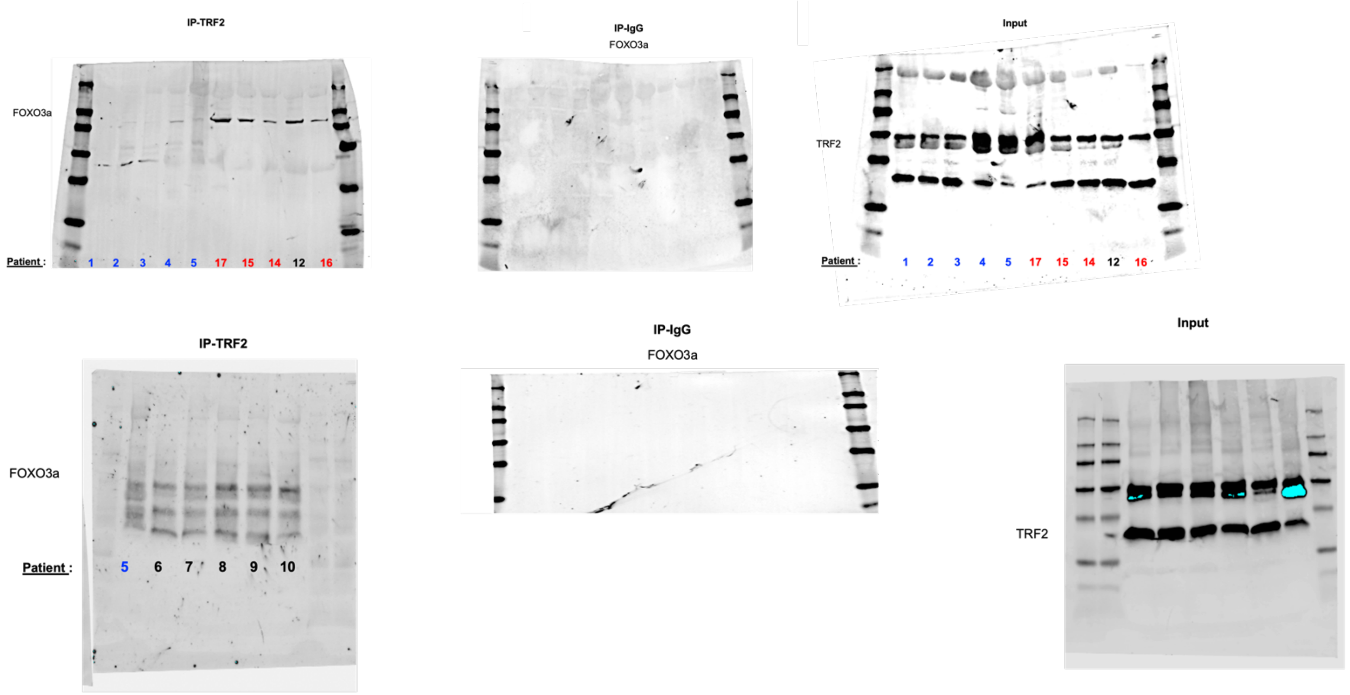

Supplemental Figure 11

Uncropped blot Supplemental Fig. 4a

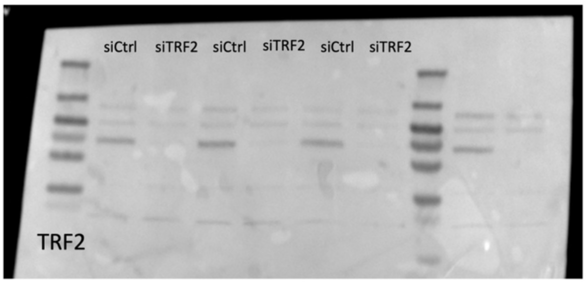

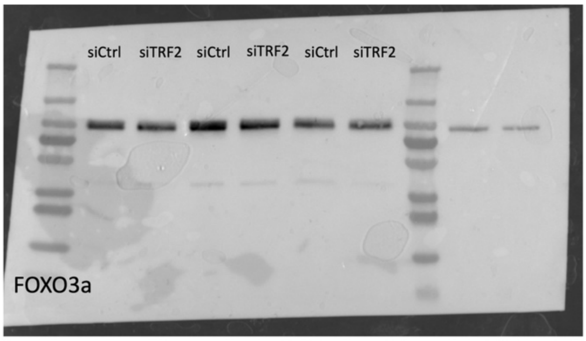

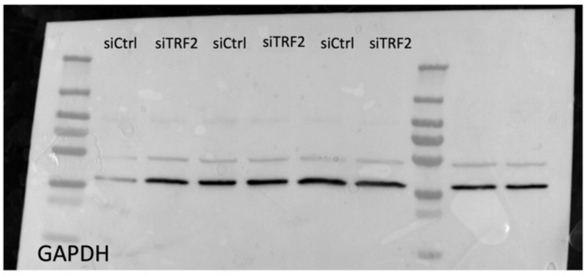

Supplemental Figure 12

Uncropped blot Supplemental Fig. 4g

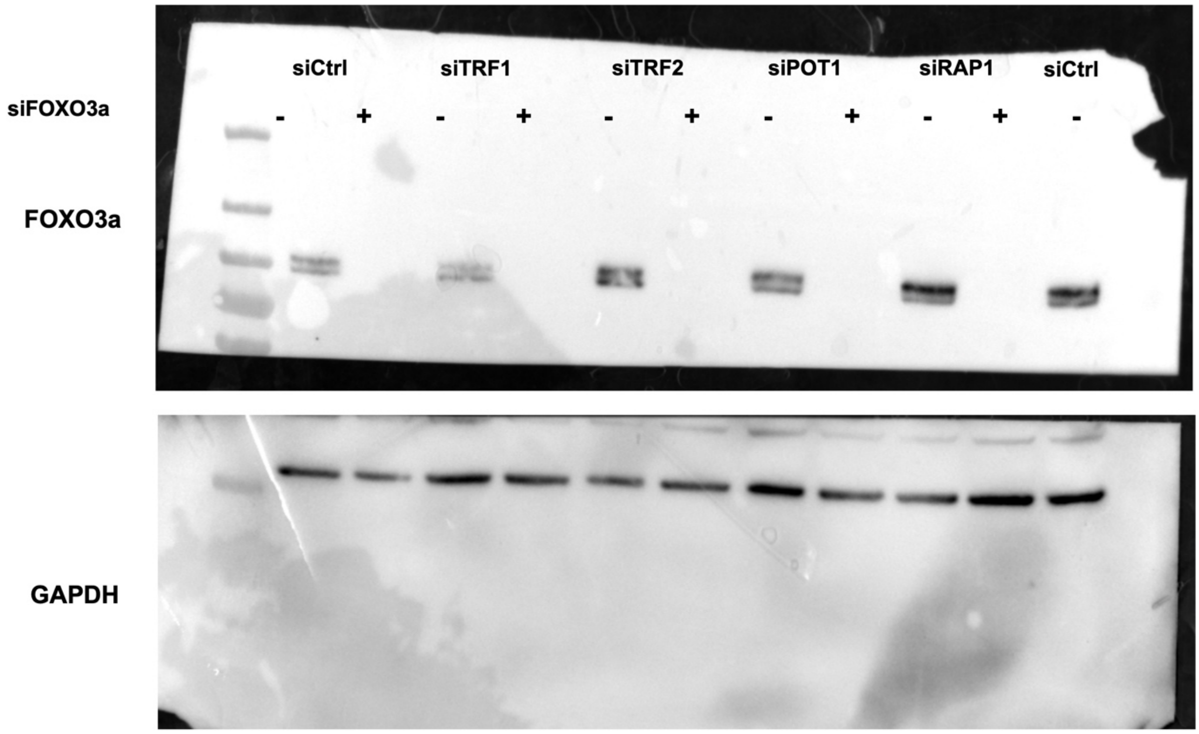

Supplement: Supplementary file 2 — Supplementary Information [file 42003_2023_4903_MOESM2_ESM.pdf]
