# Supplementary material for: Non-canonical telomere protection role of FOXO3a of human skeletal muscle cells regulated by the TRF2-redox axis
Source: Commun Biol. 2023 May 25;6:561. doi: 10.1038/s42003-023-04903-1 (PMC10213016; doi:10.1038/s42003-023-04903-1)
Supplement: Supplementary file 5 — Reporting Summary [file 42003_2023_4903_MOESM5_ESM.pdf]

nature portfolio

Corresponding author(s): Eric Gilson

Last updated by author(s): Jul 17, 2022

Reporting Summary

Nature Portfolio wishes to improve the reproducibility of the work that we publish. This form provides structure for consistency and transparency in reporting. For further information on Nature Portfolio policies, see our Editorial Policies and the Editorial Policy Checklist.

Statistics

For all statistical analyses, confirm that the following items are present in the figure legend, table legend, main text, or Methods section.

n/a	Confirmed	
☐	☒	The exact sample size (*n*) for each experimental group/condition, given as a discrete number and unit of measurement
☐	☒	A statement on whether measurements were taken from distinct samples or whether the same sample was measured repeatedly
☐	☒	The statistical test(s) used AND whether they are one- or two-sided<br>*Only common tests should be described solely by name; describe more complex techniques in the Methods section.*
☒	☐	A description of all covariates tested
☐	☒	A description of any assumptions or corrections, such as tests of normality and adjustment for multiple comparisons
☐	☒	A full description of the statistical parameters including central tendency (e.g. means) or other basic estimates (e.g. regression coefficient) AND variation (e.g. standard deviation) or associated estimates of uncertainty (e.g. confidence intervals)
☐	☒	For null hypothesis testing, the test statistic (e.g. *F*, *t*, *r*) with confidence intervals, effect sizes, degrees of freedom and *P* value noted<br>*Give P values as exact values whenever suitable.*
☒	☐	For Bayesian analysis, information on the choice of priors and Markov chain Monte Carlo settings
☒	☐	For hierarchical and complex designs, identification of the appropriate level for tests and full reporting of outcomes
☐	☒	Estimates of effect sizes (e.g. Cohen's *d*, Pearson's *r*), indicating how they were calculated

Our web collection on statistics for biologists contains articles on many of the points above.

Software and code

Policy information about availability of computer code

Data collection	*Provide a description of all commercial, open source and custom code used to collect the data in this study, specifying the version used OR state that no software was used.*
Data analysis	GraphPad/ Prism v7

For manuscripts utilizing custom algorithms or software that are central to the research but not yet described in published literature, software must be made available to editors and reviewers. We strongly encourage code deposition in a community repository (e.g. GitHub). See the Nature Portfolio guidelines for submitting code & software for further information.

Data

Policy information about availability of data

All manuscripts must include a data availability statement. This statement should provide the following information, where applicable:

- Accession codes, unique identifiers, or web links for publicly available datasets
- A description of any restrictions on data availability
- For clinical datasets or third party data, please ensure that the statement adheres to our policy

The collection of fetal muscle biopsies was approved by the “Agence Française de la Biomedecine” of the Ministery of Health for legal access to the biological material in full accordance with the law (research protocol number PFS13-006). Samples were obtained after therapeutic abortion. Parents have provided written informed consent for the use of biopsies for medical research in accordance with the Declaration of Helsinki. Muscle biopsies were processed by fetopathologists from fetuses not affected by a muscular pathology. Skeletal muscle biopsies from teens and adults were obtained from the Nice Hospital (CHU l’Archet registered as

protocol number DC-2015 2374) and from the Tumorothèque, Assistance Publique des Hôpitaux de Marseille, agreement n°AC-2013-1786, from healthy donors using standardized muscle biopsy protocol.

Human research participants

Policy information about studies involving human research participants and Sex and Gender in Research.

Reporting on sex and gender	Skeletal muscle biopsies were collected from male and female donors.
Population characteristics	Fetal, teenagers and adults participants.
Recruitment	The collection of fetal muscle biopsies was approved by the "Agence Française de la Biomedecine" of the Ministery of Health for legal access to the biological material in full accordance with the law (research protocol number PFS13-006). Samples were obtained after therapeutic abortion. Parents have provided written informed consent for the use of biopsies for medical research in accordance with the Declaration of Helsinki. Muscle biopsies were processed by fetopathologists from fetuses not affected by a muscular pathology. Skeletal muscle biopsies from teens and adults were obtained from the Nice Hospital (CHU l'Archet registered as protocol number DC-2015 2374) and from the Tumorothèque, Assistance Publique des Hôpitaux de Marseille, agreement n°AC-2013-1786, from healthy donors using standardized muscle biopsy protocol.
Ethics oversight	CHU l'Archet registered as protocol number DC-2015 2374 and Tumorothèque, Assistance Publique des Hôpitaux de Marseille, agreement n°AC-2013-1786

Note that full information on the approval of the study protocol must also be provided in the manuscript.

Field-specific reporting

Please select the one below that is the best fit for your research. If you are not sure, read the appropriate sections before making your selection.

☒ Life sciences ☐ Behavioural & social sciences ☐ Ecological, evolutionary & environmental sciences

For a reference copy of the document with all sections, see nature.com/documents/nr-reporting-summary-flat.pdf

Life sciences study design

All studies must disclose on these points even when the disclosure is negative.

Sample size	For microscopy assays, at least 30 cells were analyzed per condition and per replicate. Images were obtained from independent replicates an were randomly taken all over the slide area.
Data exclusions	Any data was excluded
Replication	Experiments were repeated at least twice. Each replicate is independent. For downregulation and overexpression assays, gene expression was checked for each replicate.
Randomization	Images were randomly taken.
Blinding	*Describe whether the investigators were blinded to group allocation during data collection and/or analysis. If blinding was not possible, describe why OR explain why blinding was not relevant to your study.*

Reporting for specific materials, systems and methods

We require information from authors about some types of materials, experimental systems and methods used in many studies. Here, indicate whether each material, system or method listed is relevant to your study. If you are not sure if a list item applies to your research, read the appropriate section before selecting a response.

Materials & experimental systems

n/a	Involved in the study	
☐	☒	Antibodies
☐	☒	Eukaryotic cell lines
☒	☐	Palaeontology and archaeology
☒	☐	Animals and other organisms
☒	☐	Clinical data
☒	☐	Dual use research of concern

Methods

n/a	Involved in the study	
☒	☐	ChIP-seq
☒	☐	Flow cytometry
☒	☐	MRI-based neuroimaging

Antibodies

Antibodies used	FOXO3a, rabbit monoclonal (clone 75D8, 1:200, Cell Signaling Technology)<br>53BP1, rabbit polyclonal (NB 100-305, 1:200, Novus Biologicals)<br>TRF2, mouse monoclonal, (NB100-56506, 1:300, Novus Biologicals)<br>TRF2, rabbit monoclonal, (NB110-57130, 1:5000, Novus Biologicals)<br>GAPDH, rabbit polyclonal (1:2000, NB100-56875, Novus Biologicals)
Validation	All these antibodies validated in human cells according to manufacturer's website.

Eukaryotic cell lines

Policy information about cell lines and Sex and Gender in Research

Cell line source(s)	Human myoblasts in this study were isolated from a 27 years old female donor (left deltoïd).<br><br>BJ-HELT (neonatal male foreskin cells) were immortalized by transduction of hTERT and SV40 ER genes.
Authentication	None of the used cell lines were authenticated
Mycoplasma contamination	Both cell lines were tested negative for mycoplasma
Commonly misidentified lines (See ICLAC register)	*Name any commonly misidentified cell lines used in the study and provide a rationale for their use.*
