# Supplementary material for: Non-canonical telomere protection role of FOXO3a of human skeletal muscle cells regulated by the TRF2-redox axis
Source: Commun Biol. 2023 May 25;6:561. doi: 10.1038/s42003-023-04903-1 (PMC10213016; doi:10.1038/s42003-023-04903-1)
Supplement: Supplementary file 1 — Gilson_Peer Review File [file 42003_2023_4903_MOESM1_ESM.pdf]

nature portfolio

Peer Review File

Non-canonical telomere protection role of FOXO3a of human skeletal muscle cells regulated by the TRF2-redox axis

Open Access This file is licensed under a Creative Commons Attribution 4.0 International License, which permits use, sharing, adaptation, distribution and reproduction in any medium or format, as long as you give appropriate credit to the original author(s) and the source, provide a link to the Creative Commons license, and indicate if changes were made. In the cases where the authors are anonymous, such as is the case for the reports of anonymous peer reviewers, author attribution should be to 'Anonymous Referee' followed by a clear attribution to the source work. The images or other third party material in this file are included in the article's Creative Commons license, unless indicated otherwise in a credit line to the material. If material is not included in the article's Creative Commons license and your intended use is not permitted by statutory regulation or exceeds the permitted use, you will need to obtain permission directly from the copyright holder. To view a copy of this license, visit http://creativecommons.org/licenses/by/4.0/.

Reviewers' comments:

Reviewer #1 (Remarks to the Author):

A follow-up study to the group's Aging Cell paper, where they showed TRF2 deletion in non-dividing myotubes does not induced telomere DDR (TIFFs), but oxidative stress and activates FOXO3a. This is overall a nice paper, if a bit narrow in scope. Figures 1 and 2 seem fine, but figure 3 has a significant technical concern in regards to PLA and CR1 mutant expression which will be described below.
For all figures:
• All data points should be shown in bar graphs
• In general, standard error of the mean is uninformative for this type of data. Standard deviation or 95% confidence interval would be better.
• In several cases t-tests are used with data sets that contain more than 2 comparisons, and this is not appropriate. In several instances the statistical test used is not mentioned (Fig 2B for example)

Figure 1:
• A schematic of the treatments would be helpful to start the figure
• Scale bar description missing from figure/legend.
• Individual channels (at lease 53BP1) should be shown in addition to merge
• Stats are missing for 1F
• The stats for 1H are not described, and stats on an n of 2 is inappropriate
• The authors show in this paper and their previous one that TRF2kd induces ROS, but no where is it mentioned what kind of ROS is being detected, including the figure legend and methods.
• The authors state in the text that "the expression of a selected well-known panel of oxidative stress-related genes (e.g., NRF2, GSS, BNIP3, 126 PRDX1, SOD2, and CAT) was not altered by FOXO3a downregulation in TERF2- compromised myotubes (Supp. Figure 1G-L)." However, SOD2 and CAT are significantly down-regulated with FOXO3a KD, and CAT is down with TERF2kd. This needs to be corrected and clarified.
• It is unclear what the difference between Figure 1B and Supp Fig 1A is.
• Since the authors make a point of the telomere specificity for damage with FOXO3a and TRF2kd, the total number of 53BP1 foci per cell should be shown for Figure 1C
• Use of PLA alongside IF/FISH is great to show FOXO3a localization. However, the PLA controls (no antibody) are missing here.
• For supplemental 2C, there are no input controls or IgG antibody controls for the pull-downs. This is unacceptable
Figure 2:
• In line 163, the authors state FOXO3akd did not induce TIFFs, but TRF2kd did, however this is not what the graph in 2B shows
• For 2D, is the siFOXO3a NT vs Bleo statistically different?
• Representative images should be included for Fig 2D UV and starved
• The authors claim "Only bleomycin-treated cells showed an effect of FOXO3a downregulation on telomere protection" based on the increase in TIFFS compared to siControl. However, this is likely due to the total number of 53BP1 foci being huge with bleomycin. For transparency and clarity, the authors should report the percent or number of telomeres colocalizing with 53BP1 for this experiment.
• The results looking at FOXO3a localization after damage are interesting and combined with the FOXO3/TRF2 PLA support a role for foxo3a going to telomeres after damage,
o However, this needs to be compared to UV. Serum starvation does not produce DNA damage comparable to UV or bleo
• Use of PLA controls here is appreciated
• In supplemental 3B the y axis says TERF2 but should be foxo3a
• The authors speculate the reason they do not see foxo3a relocalization in the BJ HELT cells is

because of the SV40 transformation. Comparing these cells to regular hTERT BJ seems an obvious way to check this

• Related to the above point, if FOXO3a is protecting telomeres in these dividing cells when TRF2 is knocked down or with bleomycin, why isn't it relocalizing? The authors should do PLA or IF/FISH in these conditions

o In the discussion (lines 263-269) the authors further comment on this saying the bleo induced translocation was less than starved, even though PLA increased. Another reason to check another type of DNA damage like UV

Figure 3:

• The authors state the CR1 mutant cannot be recognized by the cell signaling antibody used for most experiments. However, the methods section indicates the rescue vectors have GFP tags, so the authors must show expression this way. Otherwise the CR1 mutant data cannot be interpreted. In the supplement they use a different antibody which they say cannot recognize CR3, but still CR1 protein is not detected. There is no evidence it is expressed.

• Typo (endogen) in figure 3b legend

• How can the authors draw conclusions on foxo3a CR1-trf2 PLA using the cell signaling antibody as the methods state, but they say (and show in the western in fig 3b) this antibody does not recognize CR1 mutant??????

o Moreover, these PLA images show a lot of non-nuclear signal, which leads me to have little confidence in this data considering the above point

• At least as far as TIFFs go, the effect of CR2 mutation seems sound

Reviewer #2 (Remarks to the Author):

The current manuscript by Jacome Burbano et al entitled "Non-canonical role of FOXO3a in protection of the telomeres of human skeletal muscle cells regulated by the TRF2-redox axis" reports a transcription-independent role of FOXO3 binding to telomeres and thereby protecting them against ATM activation. The manuscript is timely, well-written and with intriguing data which are of interest for the readers of "Communications Biology".

The major concern regarding the data is that they did not convincingly prove that the effect is due to direct FOXO3 telomere interaction and not due to a transcriptional effect of FOXO3 or the interaction with other factors such as ATM.

There is no "Materials and Methods" section in the provided pdf file.

It is not clear whether "upon TERF2 inhibition" refers and it should be mentioned that this is shRNA-mediated and shown in Suppl Fig 1C. has there been used a second independent sequence to silence TERF2?

It should be demonstrated that N-acetylcysteine (NAC) and epigallocatechin gallate (EGCG) effectively reduce oxidative stress in the cellular model used.

It is not clear to what type of activation the statement "activates the longevity factor FOXO3a" refers to? Expression, nuclear localization, transcriptional activity?

Correct typo in "These results sshow"

Figure 1 E. claimed nuclear translocation of FOXO3 is not visible. This would be an important piece of data as this would prove that the antibody really detects FOXO3. Colocalization is not convincing as FOXO3 expression increases upon TERF2 silencing, increasing the number of dots, some of them

overlap with the green dots.

Figure 1G. The meaning of the two red spots is not clear and image quality does not allow for evaluation of these data.

Figure 2D Bleomycin induced increase in telomere damage as shown in this figure is not convincing. No description of the quantification method has been provided.

Reviewer #3 (Remarks to the Author):

This is an interesting manuscript that deserves publication upon few additional controls.

Fig 1A, B. It would be good to have a sense of the efficacy of the antioxidant treatments by showing reduced ROS levels under the experimental settings used.

Fig1e,f: is the difference observed statistically significant?

Suppl 2B needs quantification

The observed TRF2-FOXO3a interaction during aging is unexpected and interesting: are the levels of FOXO3a regulated similarly to TRF2 during aging?

Given the reported role of FOXO3A in activating ATM, rather than muting it as reported here, and given FOXO3a role in aging in many species, it would be interesting to discuss any data from the literature on the impact of mutations in the CR2C domain (or other domains) on the lifespan of animal models and/or if SNPs in such domain have been studied in correlation with lifespan in humans.

Author responses to reviewers' comments

Reviewers' comments:

Reviewer #1 (Remarks to the Author):

A follow-up study to the group's Aging Cell paper, where they showed TRF2 deletion in non-dividing myotubes does not induced telomere DDR (TIFFs), but oxidative stress and activates FOXO3a. This is overall a nice paper, if a bit narrow in scope. Figures 1 and 2 seem fine, but figure 3 has a significant technical concern in regards to PLA and CR1 mutant expression which will be described below.
For all figures:
• All data points should be shown in bar graphs
• In general, standard error of the mean is uninformative for this type of data. Standard deviation or 95% confidence interval would be better.
• In several cases t-tests are used with data sets that contain more than 2 comparisons, and this is not appropriate. In several instances the statistical test used is not mentioned (Fig 2B for example)

We thank the reviewer for his/ her advice. In the modified manuscript, all graphs have been modified to show the mean of each replicate. Besides, statistical description has been checked and added in all the figures. However, we should mention that for some figures t-tests are used to compare data two by two because they correspond to two different biological conditions: for example, siControl and siFOXO3a, plus a genotoxic treatment. In this case we compare if the treatment has an effect on siControl transfected cells and next we compare whether the treatment has a different response when FOXO3a is downregulated.

Figure 1:
• A schematic of the treatments would be helpful to start the figure

The following scheme has been added to introduce Figure 1.

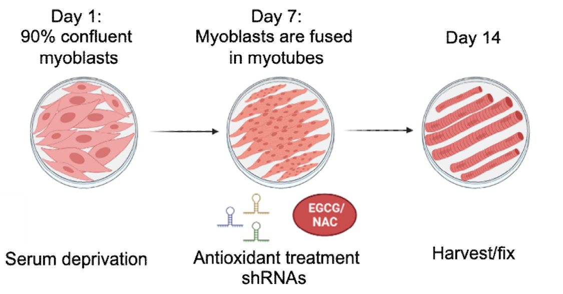

• Scale bar description missing from figure/legend.

We apologize for this oversight; scale bar description has been added in the revised manuscript figure legends.

• Individual channels (at lease 53BP1) should be shown in addition to merge

A new panel showing 53BP1 and merge have been added in old figure 1A, new figure 1B.

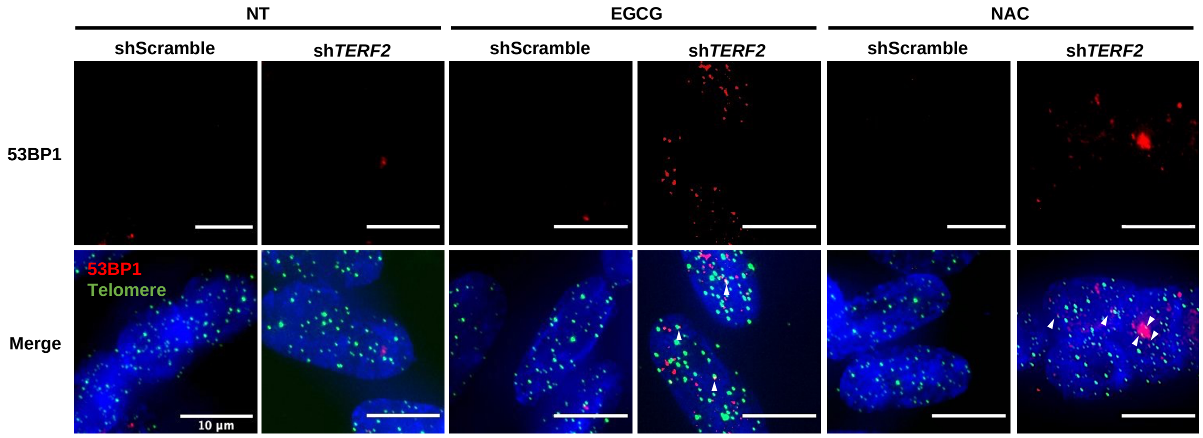

• Stats are missing for 1F

We thank the reviewer for rising up this oversight. Statistical analysis has been added.

• The stats for 1H are not described, and stats on an n of 2 is inappropriate

We have completed our data in order to show triplicates for figure 1H (new figure 1).

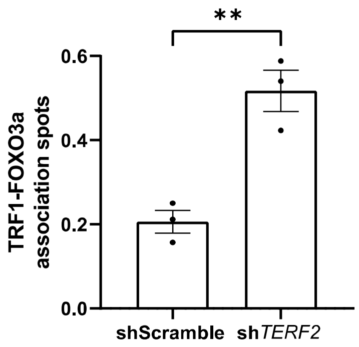

• The authors show in this paper and their previous one that TRF2kd induces ROS, but no where is it mentioned what kind of ROS is being detected, including the figure legend and methods.

We apologize for this oversight. In both papers, we used Enzo kit (ROS-ID® Total ROS detection kit: ENZ-51011). According to manufacturer description, the dye reacts with reactive species (reactive oxygen and nitrogen species). However, it does not recognize superoxides. To clarify this point, the material and methods section has been modified as it follows:

« Cells were grown on coverslips and treated as indicated in the manufacturer's ROS kit instructions (Enzo Enz-51011) and previously described. This kit recognizes different reactive species (hydroxyl radicals, hydrogen peroxide, peroxynitrite) but does not detect superoxide. »

• The authors state in the text that "the expression of a selected well-known panel of oxidative stress-related genes (e.g., NRF2, GSS, BNIP3, 126 PRDX1, SOD2, and CAT) was not altered by FOXO3a downregulation in TERF2- compromised myotubes (Supp. Figure 1G-L)." However, SOD2 and CAT are

significantly down-regulated with FOXO3a KD, and CAT is down with TERF2kd. This needs to be corrected and clarified.

We thank the reviewer for pointing this out. In this figure we wanted to determine whether a differential regulation of genes related in the oxidative stress response could explain the presence of damage at telomeric DNA in *TERF2* and *FOXO3a*-compromised myotubes. As the reviewer mentioned, catalase is downregulated in sh*TERF2*, sh*FOXO3a* myotubes and in the double knockdown. Likewise, SOD2 is downregulated in a FOXO3a-dependent manner. Nevertheless, these gene regulations are not significantly affected in the double downregulation of FOXO3a and TERF2, compared to the single gene downregulation condition. Hence, our results suggest telomere deprotection observed in the double *FOXO3a* and *TERF2* downregulation cannot be explained by a decrease in the oxidative-stress response. This point will be clarified in the revised version as it follows:

"Moreover, we studied the expression of a well-known panel of oxidative stress-related genes (e.g., *NRF2*, *GSS*, *BNIP3*, *PRDX1*, *SOD2*, and *CAT*). Among them, *CAT* and *SOD2* were downregulated upon *FOXO3A* KD but not further repressed in myotubes doubly downregulated for *TERF2* and *FOXO3a* (Supp. Figure 2c-h)."

• It is unclear what the difference between Figure 1B and Supp Fig 1A is.

We thank the reviewer for his/her comment. Indeed, we did a mistake when labelling the axis. Supp Fig 1A corresponds to the number of TIFs, and in Figure 1c (previously Figure 1B) it is the telomeric specific damage. Graph axis title has been corrected.

• Since the authors make a point of the telomere specificity for damage with FOXO3a and TRF2kd, the total number of 53BP1 foci per cell should be shown for Figure 1C

As the reviewer requested, we added the number of TIFs and 53BP1 foci per cell in Supp. Figure 1d-e

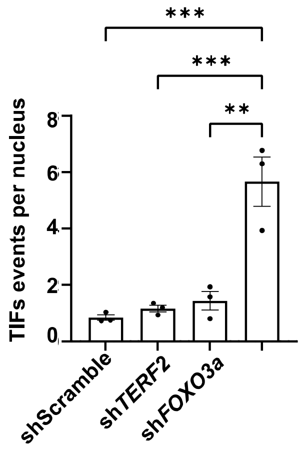

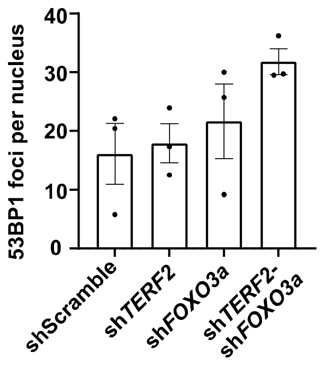

• Use of PLA alongside IF/FISH is great to show FOXO3a localization. However, the PLA controls (no antibody) are missing here.

We thank he reviewer for his/her valuable remark. In the revised manuscript the missing control was added (Supplemental Figure 1G and H). Three negative controls were carried out: PLA staining using exclusively i) PLA probe, ii) FOXO3a or iii) TRF1 antibody. Whereas, negative controls showed slightly

lower PLA spots than in shScramble condition, there was a significant increase in *TERF2* downregulated myotubes.

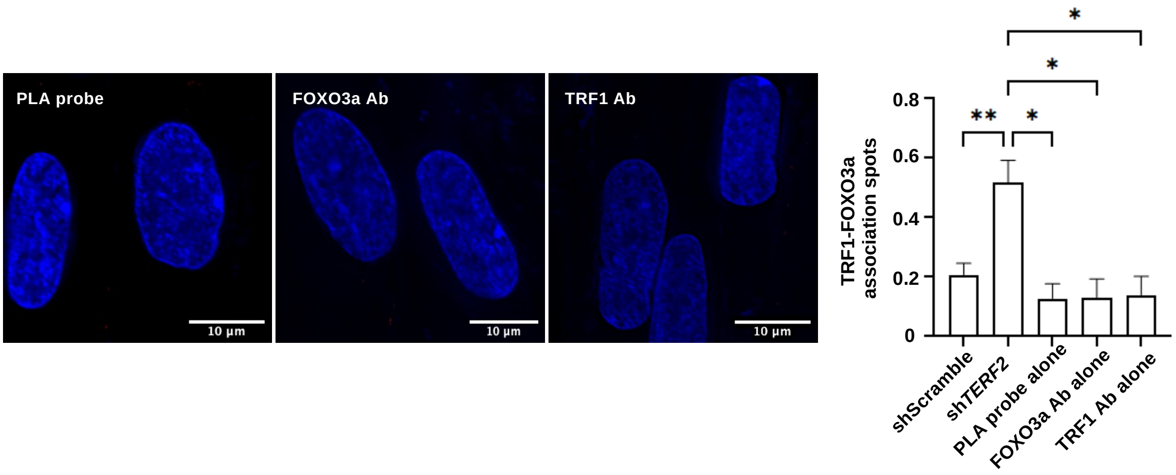

• For supplemental 2C, there are no input controls or IgG antibody controls for the pull-downs. This is unacceptable

We thank the reviewer for this pertinent remark regarding this mislabeled figure. We have modified the supplemental figure 2C accordingly to avoid any confusion. For each condition, we present WBs using a FOXO3a antibody after IP using either TRF2 or IgG antibodies; respectively. Our assay was performed using samples normalized to TRF2 protein levels (estimated) and reported in the last WB labeled Input, TRF2.

We provide below a complementary figure that includes additional controls.

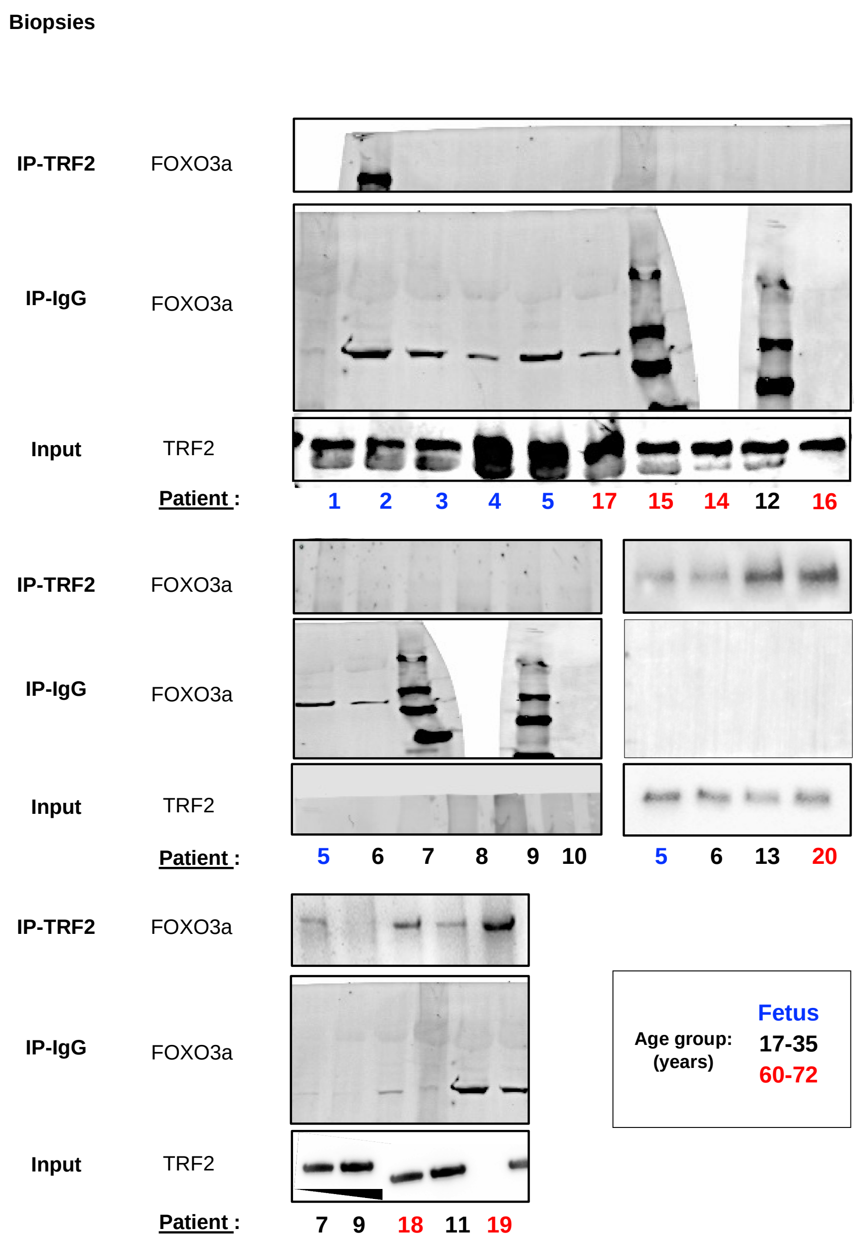

Figure 2:

• In line 163, the authors state FOXO3akd did not induce TIFFs, but TRF2kd did, however this is not what the graph in 2B shows

We thank the reviewer for raising this important mistake. Indeed, the name of si*TERF2* and si*FOXO3a* were inverted. It has been corrected on the revised version.

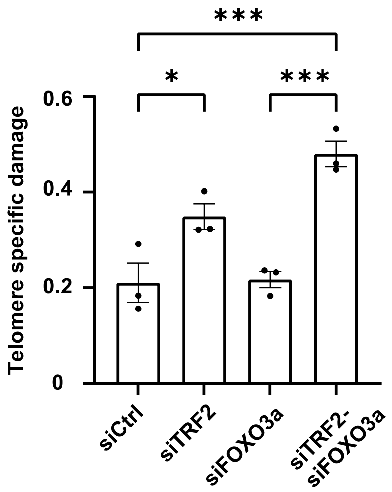

• For 2D, is the siFOXO3a NT vs Bleo statistically different?

We thank the reviewer for his/her observation. Statistical analyses were completed in the revised version. siFOXO3a-Bleo shows higher specific damage compared to siFOXO3a-NT (Figure 3B). Our results were further supported by the percentage of damaged telomeres (e.g., percentage of telomeres colocalizing with 53BP1) (Figure 3C).

Figure 3B

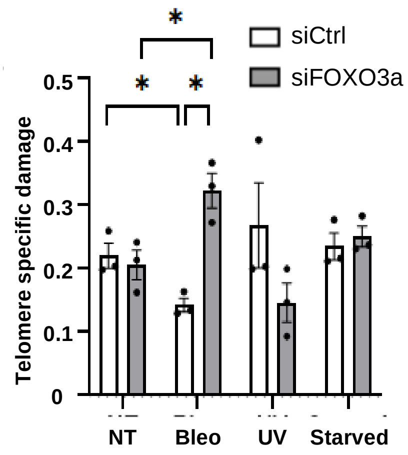

Figure 3c

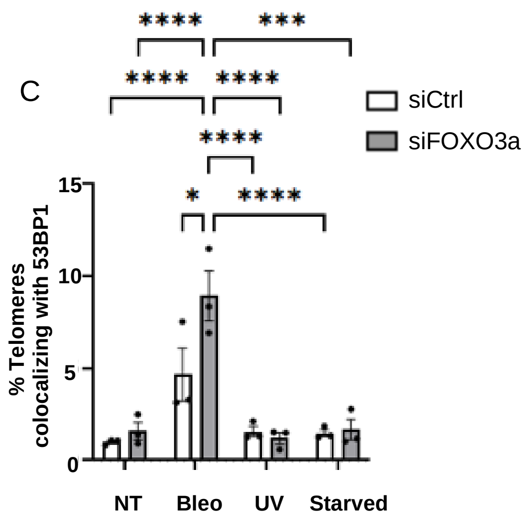

• Representative images should be included for Fig 2D UV and starved

The figure panel was completed with UV and starved images in Fig 3d.

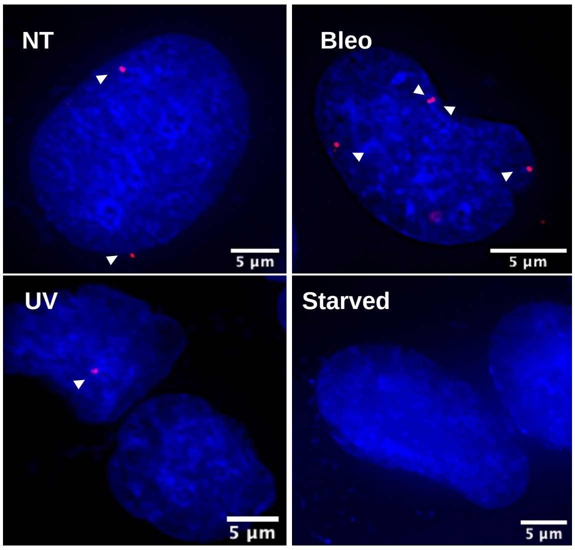

• The authors claim “Only bleomycin-treated cells showed an effect of FOXO3a downregulation on telomere protection” based on the increase in TIFFS compared to siControl. However, this is likely due to the total number of 53BP1 foci being huge with bleomycin. For transparency and clarity, the authors should report the percent or number of telomeres colocalizing with 53BP1 for this experiment.

We agree that showing the percentage of damaged telomeres would enhance the clarity of the results. Therefore, we added this value in Fig 3c. In our system, BJ-HELT cells exposed to UV or starvation showed around 2% damaged telomeres, similar to non-treated cells. Nevertheless, 24h bleomycin treatment led to a 2-fold increase and this damage was exacerbated under siFOXO3a condition (4-fold increase).

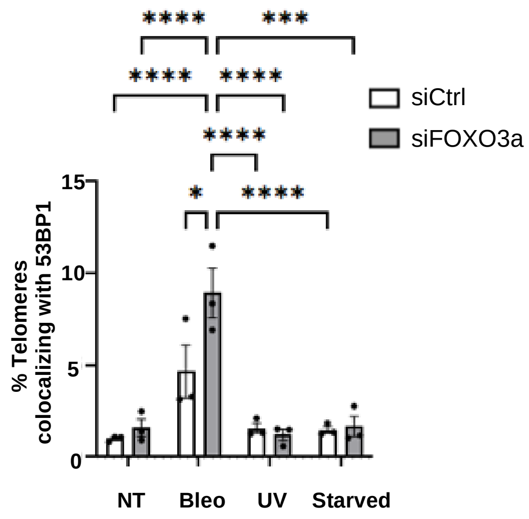

• The results looking at FOXO3a localization after damage are interesting and combined with the FOXO3/TRF2 PLA support a role for foxo3a going to telomeres after damage,
o However, this needs to be compared to UV. Serum starvation does not produce DNA damage comparable to UV or bleo

We agree with the reviewer that this data was missing. Hence, we performed TRF2-FOXO3a PLA in BJ-HELT cells exposed to UV. However, UV did not trigger FOXO3a and TRF2 association. (Fig. 3d and e).

• Use of PLA controls here is appreciated
• In supplemental 3B the y axis says TERF2 but should be foxo3a

We apologize for this mistake. It has been corrected in the new manuscript.

• The authors speculate the reason they do not see foxo3a relocalization in the BJ HELT cells is because of the SV40 transformation. Comparing these cells to regular hTERT BJ seems an obvious way to check this

We thank reviewer for his/her comment. In order to answer to this point, we used BJ primary human fibroblasts, in which TRF2 was downregulated and FOXO3a cellular localization was studied by immunofluorescence. Three biological replicates with at least 30 cells per condition were analyzed.

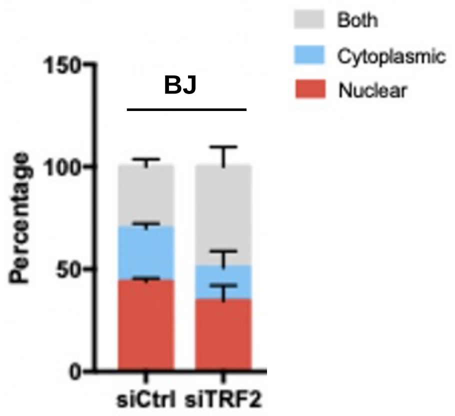

As in BJ-HELT, we found that FOXO3a is highly active in BJ cells as observed by an elevated percentage of cells containing mainly nuclear FOXO3a (30-47%) and remains unchanged upon TRF2 downregulation. Hence, our hypothesis that SV40 transformation might explain an important basal activation of FOXO3a is not correct. Thus, BJ and BJ-HELT cells display an important FOXO3a activation in unstressed conditions. The reason of this Foxo3A activation in the BJ cell line is unknown and is out of the scope of this work.

Consequently, we modified the revised manuscript as it follows: “However, in contrast to myotubes, we failed to detect a difference in FOXO3a activation upon *TERF2* downregulation in BJ-HELT cells (Supp. Figure 4d, e). Noteworthy, the unchallenged BJ-HELT cells exhibit a high level of FOXO3a in the nucleus. Thus, upon *TERF2* downregulation, the increased localization of FOXO3a at telomeres in the absence of apparent FOXO3a activation suggests that a fraction of the important pool of nuclear FOXO3a existing in BJ-HELT cells can be mobilized to telomeres. The reason for the high level of FOXO3a activation in BJ-HELT cells is unknown.”

• Related to the above point, if FOXO3a is protecting telomeres in these dividing cells when TRF2 is knocked down or with bleomycin, why isn’t it relocalizing? The authors should do PLA or IF/FISH in these conditions

We thank reviewer for his/her remark. In order to determine how FOXO3a specifically protect telomeres, we performed a PLA between FOXO3a and TRF1 in BJHELT cells downregulated for TRF2. Our results suggest that in absence of TRF2, FOXO3a binding to telomeres is slightly but significantly increased. This observation is similar to the process we detected in TRF2 KO myotubes.

Interestingly, we failed to observe higher FOXO3a nuclear translocation when TRF2 is downregulated in BJHELT cells (Supplemental Fig. 4d-e). Therefore, we can imagine a portion of the nuclear FOXO3a is relocated at telomeres in a telomere stress context.

These results have been added in Figure 2e-f of the revised manuscript.

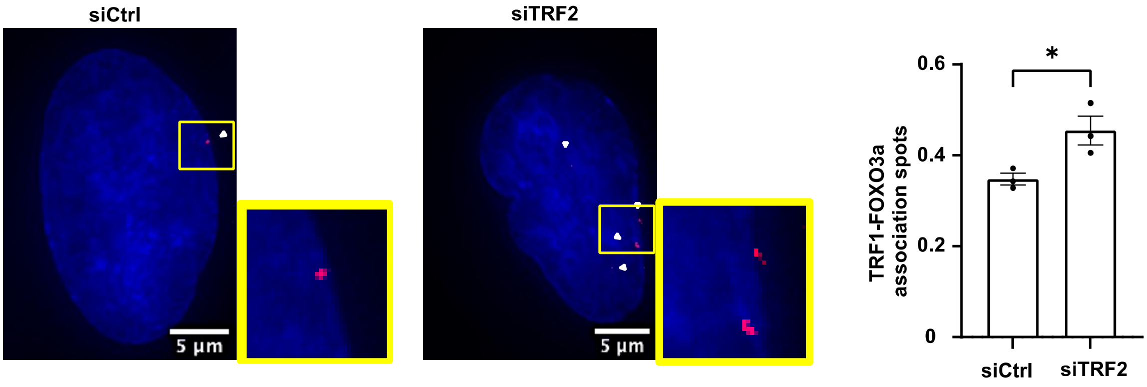

o In the discussion (lines 263-269) the authors further comment on this saying the bleo induced translocation was less than starved, even though PLA increased. Another reason to check another type of DNA damage like UV

According to the reviewer suggestion, we measured FOXO3a-TRF2 association by PLA assay. Nevertheless, in contrast to 24h bleomycin treatment, 2h after 300 mJ/cm^2 UV exposure did not induce an increased TRF2-FOXO3a association. Nevertheless, UV exposure did not trigger specific telomere damage in these conditions, neither in siControl nor in siFOXO3a samples.

It has been described that some cells are more sensitive than others to UV-induced DNA damage (Lopez Perez R., at al. 2019). Several protocols describe UV induced DNA damage after UVB exposure at 4-100 mJ/cm^2. These damages could be observed 30 min after irradiation for short term damage, or some days later for viability and proliferation assays (Lopez Perez R., at al. 2019; Lisby S, et al., 2005). Nevertheless, in our experimental set-up conditions (cell fixation 2h after 300 mJ/cm^2 UVA and UVB radiation), we did not observe specific telomeric DNA damage. We did not notice a major cell mortality after UV exposure that could explain that only undamaged cells were still alive and used for our experiments.

While surprising, studies in BJ and BJ-hTERT fibroblasts showed telomeres are partially protected from cyclobutane pyrimidine dimers (CPD) and pyrimidine (6–4) pyrimidine photoproducts (Parikh D. et al., 2015). Hence, we cannot conclude on FOXO3a-dependent telomere protective effect in UV irradiated BJ-HELT cells.

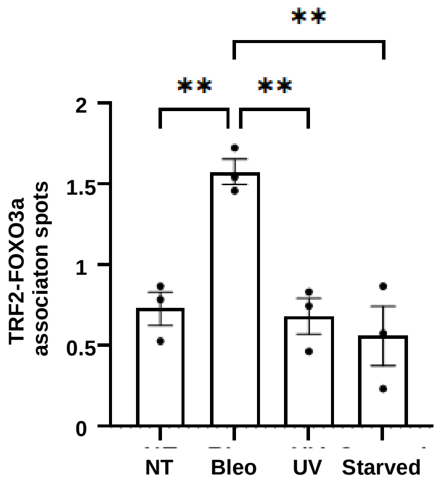

Figure 3:

• The authors state the CR1 mutant cannot be recognized by the cell signaling antibody used for most experiments. However, the methods section indicates the rescue vectors have GFP tags, so the authors must show expression this way. Otherwise the CR1 mutant data cannot be interpreted. In the supplement they use a different antibody which they say cannot recognize CR3, but still CR1 protein is not detected. There is no evidence it is expressed.

We thank the reviewer for his/ her remark. However, our viral vector does not contain a GFP-tag but a transcriptional fusion system. Therefore, an antibody against GFP would not reflect FOXO3 mutant expression.

As we are not able to compare all mutants homogenously, we decided to exclude mutant ∆CR1. We agree that we cannot conclude whether CR1 domain is involved in telomere protection function. This point has been raised in the new version's discussion by:

"Whether other FOXO3a domains than CR2C are involved in telomere protection will require further studies."

However, our data showed that neither the forkhead/ DNA binding nor the major transactivation (CR3) domain but the CR2 domain of FOXO3a that is involved in telomere protection. These results suggest that the telomeric function is independent from the transcription factor function.

• Typo (endogen) in figure 3b legend

It has been corrected in the new manuscript for "endogenous".

• How can the authors draw conclusions on foxo3a CR1-trf2 PLA using the cell signaling antibody as the methods state, but they say (and show in the western in fig 3b) this antibody does not recognize CR1 mutant??????

o Moreover, these PLA images show a lot of non-nuclear signal, which leads me to have little confidence in this data considering the above point

• At least as far as TIFFs go, the effect of CR2 mutation seems sound

We thank reviewer for giving his/her honest opinion, we agree with these critics.

The FOXO3a mutant panel in this work does not contain a tag nor are fused to GFP expression. Hence, mutant FOXO3a proteins expression can be compared exclusively using antibodies recognizing the endogenous FOXO3a. However, in the ΔCR1 mutant, the epitope recognized by the FOXO3a antibody (Cell Signalling-2497) is removed. So, this mutant cannot be compared with the others. Therefore, we decided to withdraw the CR1.

Reviewer #2 (Remarks to the Author):

The current manuscript by Jacome Burbano et al entitled “Non-canonical role of FOXO3a in protection of the telomeres of human skeletal muscle cells regulated by the TRF2-redox axis” reports a transcription-independent role of FOXO3 binding to telomeres and thereby protecting them against ATM activation. The manuscript is timely, well-written and with intriguing data which are of interest for the readers of “Communications Biology”.
The major concern regarding the data is that they did not convincingly prove that the effect is due to direct FOXO3 telomere interaction and not due to a transcriptional effect of FOXO3 or the interaction with other factors such as ATM.

We thank reviewer #2 for his/her comments.

We have several data suggesting FOXO3a is part of a telomeric complex. We have observed FOXO3a proximity association with shelterin proteins (TRF1 and TRF2), besides FOXO3a and telomeric DNA colocalization by FISH. Finally, our FOXO3a ChIP-Southern blot assay revealed that FOXO3a is linked to the telomeric DNA.

Our data showed that the neither the forkhead/ DNA binding nor the major transactivation (CR3) domain of FOXO3a are involved in telomere protection. However, our data showed that the neither the forkhead/ DNA binding nor the major transactivation (CR3) domain but the CR2 domain of FOXO3a that is involved in telomere protection. Therefore, it is unlikely that the transcriptional effect of FOXO3a is involved. Nevertheless, we cannot exclude that FOXO3a might have a transcriptional function that does not depend on the FH and transactivation domain.

Besides, ATM binds to the FOXO3a-CR3 domain. However, we cannot exclude that FOXO3a binds to other factors at telomeres (other than the shelterin complex and telomeric binding proteins).

There is no “Materials and Methods” section in the provided pdf file.

We would like to apologize. We will make sure that the Materials and Methods section is in the revised manuscript.

It is not clear whether “upon TERF2 inhibition” refers and it should be mentioned that this is shRNA-mediated and shown in Suppl Fig 1C. has there been used a second independent sequence to silence TERF2?

We have used a shRNA in myotubes and siRNA (Smart pool from Dharmacon) in BJ-HELT cells. However, we have conducted rescue experiments in both cell types. First, in myotubes downregulated for TERF2 and FOXO3a, the FOXO3a full-length form overexpression rescued the telomere protection phenotype (Figure 4F and G). Second, FOXO3a rescue in BJ-HELT fibroblasts exposed to bleomycin

restored the telomere protection, as well as the TRF2-FOXO3a association phenotype (Figure 4C and D).

It should be demonstrated that N-acetylcysteine (NAC) and epigallocatechin gallate (EGCG) effectively reduce oxidative stress in the cellular model used.

The EGCG treatment in myotubes was previously validated in Robin et al, 2020, Aging Cell. Indeed, EGCG treatment inhibited ROS accumulation, which is observed without the antioxidant treatment in sh*TERF2* myotubes.

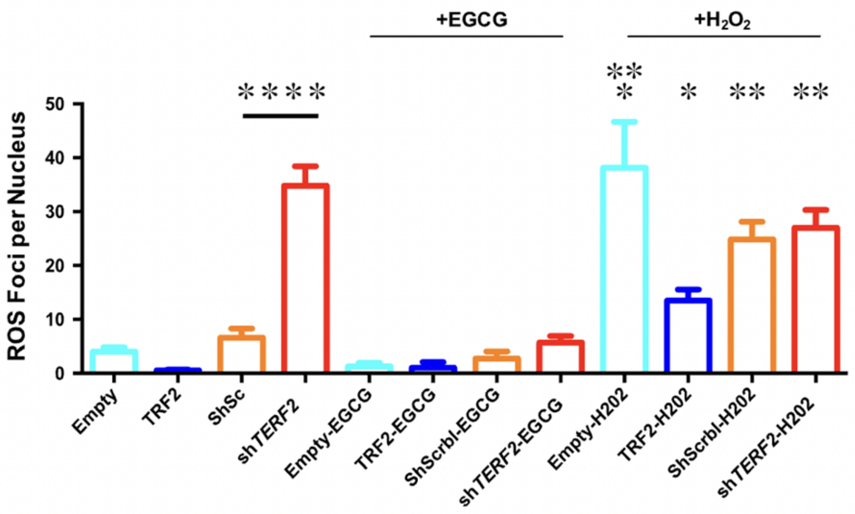

(Figure 3 *Robin et al., Aging Cell, 2020*)

It is not clear to what type of activation the statement “activates the longevity factor FOXO3a” refers to? Expression, nuclear localization, transcriptional activity?

We thank reviewer for his/her comment. When we assert that FOXO3a is activated, we refer to will its nuclear localization. This point will be clarified in the manuscript as it follows:

“activates the longevity factor FOXO3a, as assayed by its nuclear translocation”.

Correct typo in “These results sshow”

We apologize for this mistake that has been corrected in the new manuscript.

Figure 1 E. claimed nuclear translocation of FOXO3 is not visible. This would be an important piece of data as this would prove that the antibody really detects FOXO3. Colocalization is not convincing as FOXO3 expression increases upon TERF2 silencing, increasing the number of dots, some of them overlap with the green dots.

In order to provide more convincing photos, we changed our panel in Figure 1F (previous 1E). New

picture show better that in myotubes expressing TERF2, FOXO3a has a cytoplasmic localization. Moreover, we added the quantification of FOXO3a foci per nuclei in Fig. Supp. 3a.

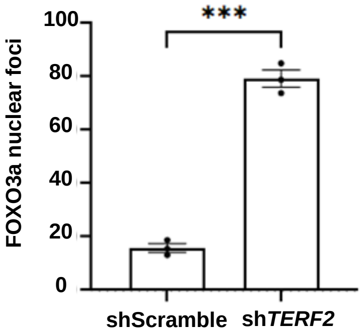

Figure 1G. The meaning of the two red spots is not clear and image quality does not allow for evaluation of these data.
The few dots in these pictures correspond to an association between TRF1 and FOXO3a. We agree that there are just a few dots. However, our results are supported by PLA negative control results, showing these antibodies specificity (Supplemental Fig.1g and h) and by a telomere-FOXO3a colocalization quantification (Figure 1f) showing an increase FOXO3a telomeric localization in the absence of *TERF2*.

Figure 2D Bleomycin induced increase in telomere damage as shown in this figure is not convincing. No description of the quantification method has been provided.

Data provided in Figure 2D correspond to the ratio between the number of damaged telomeres to the total number of 53BP1 foci.

The quantification method will be described in the revised manuscript, as it follows:

"Pictures were taken using a DeltaVision Elite system (GE). An average of 70 stacks and 40 nuclei were taken per conditions. Images were then analyzed using FIJI (ImageJ). Total number of 53BP1 foci was determine and telomere-53BP1 colocalizations (TIFs) were determined by doing a stack-by-stack screening looking for at least one colocalizing pixel (yellow).

The telomeric specific damage was calculated as the ratio between the number of TIFs and the total number of 53BP1 foci."

Reviewer #3 (Remarks to the Author):

This is an interesting manuscript that deserves publication upon few additional controls.

Fig 1A, B. It would be good to have a sense of the efficacy of the antioxidant treatments by showing reduced ROS levels under the experimental settings used.
The EGCG treatment in myotubes was previously validated in Robin et al, 2020, Aging Cell. Indeed, EGCG treatment inhibited ROS accumulation that is observed without the antioxidant treatment in sh*TERF2* transduced myotubes.

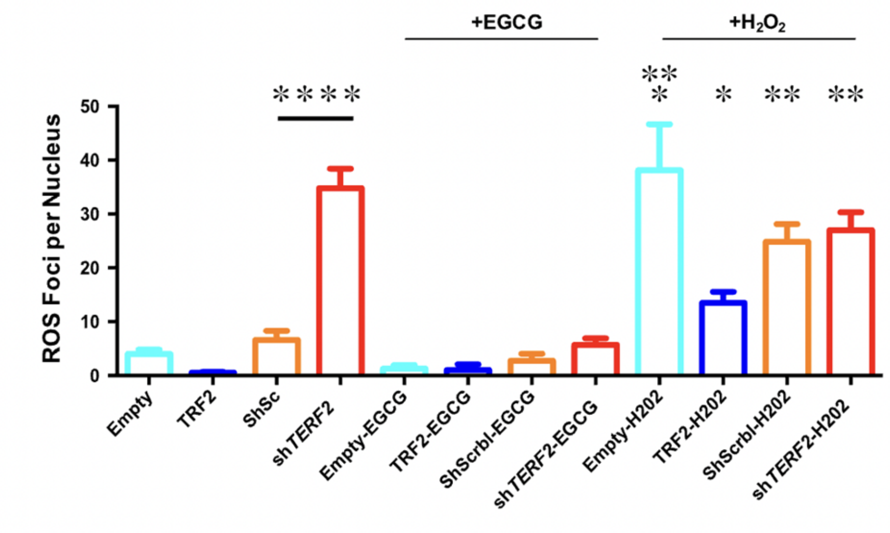

(Figure 3 *Robin et al., Aging Cell, 2020*)

Fig1e,f: is the difference observed statistically significant?

We apologize for this oversight; statistical results have been added in the revised manuscript (Figure 1g).

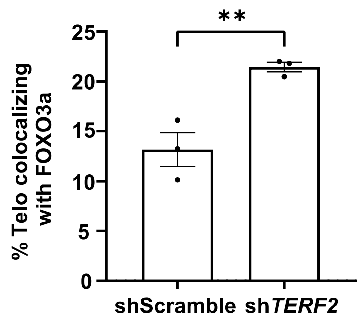

Suppl 2B needs quantification

Quantification has been added in Supplemental figure 3c.

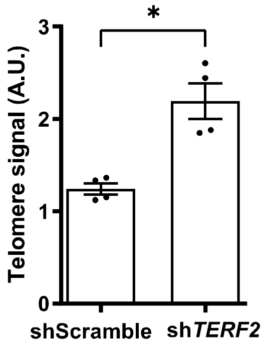

The observed TRF2-FOXO3a interaction during aging is unexpected and interesting: are the levels of FOXO3a regulated similarly to TRF2 during aging?

We thank the reviewer for his/her interesting question. This point will be address in a following project. For now, we have some data from human skeletal muscle biopsies (figure here below). However, our cohort size is too small to have confirmed results. Hence, we are glad to show this information to the reviewer but we do not think it is appropriate to add it in the revised manuscript.

Our preliminary results suggest a slight increase of FOXO3a with age but this should be confirmed with more biopsies samples.

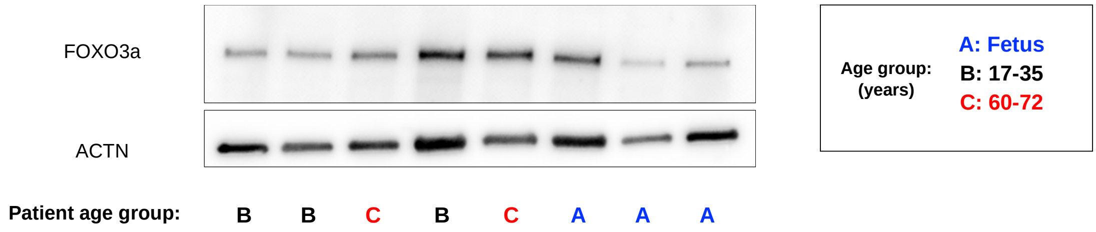

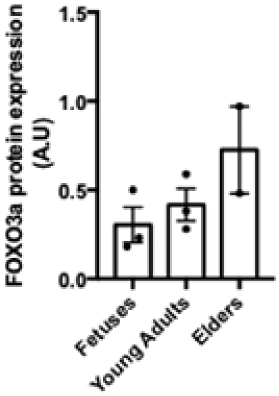

Given the reported role of FOXO3A in activating ATM, rather than muting it as reported here, and

given FOXO3a role in aging in many species, it would be interesting to discuss any data from the literature on the impact of mutations in the CR2C domain (or other domains) on the lifespan of animal models and/or if SNPs in such domain have been studied in correlation with lifespan in humans.

We thank you the reviewer for his/ her advice.
Even if FOXO3a is a protein that has been described to be involved in aging, and some SNPs are highly correlated to longevity and healthspan, these SNPs are all intronic (Sanese et al., 2019).
In this paper, we discovered that the conserved region 2C (CR2C) (461-482 aa) is required for FOXO3a telomeric association and protection. This domain, along with CR3 (483-673) are the two FOXO3a transactivation domains. The CR3 domain was shown to interact with Ataxia-telangiectasia mutated (ATM) (Tsai et al., 2008 and Chung et al., 2012). Counterintuitively, we observed that △CR3 mutant expression, increases the TRF2-FOXO3a association. Besides, the Forkhead (FH)/ DNA binding domain was not required neither for FOXO3a-telomere association neither for telomere protection. Thus, our work suggests a non-canonical FOXO3a function independent from its DNA binding domain.
Unfortunately, to our knowledge, any animal model carrying a mutation in the CR2C domain has been created or characterized yet.
FOXO3a first implications on longevity was unveiled in *C. elegans* deletion mutant *daf-16* (mu86). *daf-16* mutation inhibits the lifespan extension observed in *daf-2* worms. Interestingly, this phenotype was also described in point mutation *daf-16* worms.
FOXO3-/- mice (exon 2 is floxed) are viable but have a reduced fertility associated with early unset of ovarian problems (Castrillon DH et al., 2003). Besides, FOXO3a point mutation in the Forkhead domain (transition T to A) triggers ovarian teratomas (Youngson N.A. et al., 2011).
However, FOXO3a is required for dietary restriction induced extended lifespan in mouse and *Drosophila melanogaster*.

We propose to add the following paragraph in the revised manuscript:

"In contrast to the FH and CR3 domains, deletion of CR2C impaired the telomere-protective effect of FOXO3a. CR2C exhibits transactivation activity and mediates the association of FOXO3a with SIRT1 [51] and with CBP/p300 [52,53]. In accordance with involvement of the FOXO3a–p300 complex in telomere protection, p300, but not CBP, was associated with telomeres [54], suggesting that acetylation mediated by p300 promotes the repair of damaged telomeres. This model requires substantiation, particularly by identifying the targets of FOXO3a–p300 on telomeres. One candidate is TRF2, which contains a p300 binding site [55].
Whereas FOXO3a have been closely related to longevity in several species, as studied in deletion and point mutation, any animal model carrying a mutation in the CR2C domain has been created or characterized yet. Moreover, SNP associated to extreme longevity in humans are not carried in the CR2C sequence. FOXO3a$^{-/-}$ mice are viable but females develop ovarian problems during early adulthood. We could imagine FOXO3a $^{\Delta CR2C}$ mice might have an increased sensitivity to genotoxic insults and a premature sarcopenic phenotype."

REVIEWERS' COMMENTS:

Reviewer #1 (Remarks to the Author):

Overall the authors have addressed my concerns and made extensive changes to the manuscript that improve it. I fully support its publication as is. However, the statement about t-tests and multiple comparisons is erroneous. ANOVA and post-hoc tests are the approportionate choice when comparing multiple samples with multiple variables. I would HIGHLY recommend the authors revisit their stats (not that I believe much will change, but our field is really lax when it comes to statistics), but I will leave this to the editor.
I also fully misunderstood the expression vector system with GFP being bicistronic. I understand why the CR1 mutant could not be studied under this conditions now. I look forward to seeing this manuscript publish and congratulate the authors on a nice body of work.

Reviewer #2 (Remarks to the Author):

The authors provided a significantly improved revised version of the manuscript. The comments I have raised have been addressed.

Reviewer #3 (Remarks to the Author):

my concerns have been addressed satisfactorily

Author response to reviewer comments

Additional comment of Referee 1:
Overall the authors have addressed my concerns and made extensive changes to the manuscript that improve it. I fully support its publication as is. However, the statement about t-tests and multiple comparisons is erroneous. ANOVA and post-hoc tests are the approproportionate choice when comparing multiple samples with multiple variables. I would HIGHLY recommend the authors revisit their stats (not that I believe much will change, but our field is really lax when it comes to statistics), but I will leave this to the editor.
I also fully misunderstood the expression vector system with GFP being bicistronic. I understand why the CR1 mutant could not be studied under this conditions now. I look forward to seeing this manuscript publish and congratulate the authors on a nice body of work.

We thank the reviewer for his/her comment. We have revisited our stats. The reviewer concern involves Figure 3b. Statistics have been recalculated for this figure by using a one-way ANOVA with Tukey's post hoc test. The graph and the legend of the figure have been changed accordingly. This new stat does not change the conclusion that Foxo3a is required for telomere protection upon bleo treatment.
All our comparisons of more than two groups are analyzed using a one-way ANOVA test with Tukey's post hoc.
